# Assessment and Optimization of Force Fields for Glycine Polymorphism and Solution Properties

**DOI:** 10.1021/acs.jctc.6c00162

**Published:** 2026-04-07

**Authors:** James W. Meadows, Sharon J. Cooper, Mark A. Miller, Mark R. Wilson

**Affiliations:** Department of Chemistry, 3057 Durham University, South Road, Durham DH1 3LE, U.K.

## Abstract

Molecular dynamics studies of glycine crystal growth require a force field that accurately reproduces both solution- and crystal-phase properties for all three ambient-pressure polymorphs. However, many studies use force fields validated only for α-glycine, which often yield poor crystal properties. Here, we extensively evaluate 18 force field variants (10 OPLS and 8 GAFF) and recalibrate the parameters of the best-performing models using multiobjective Bayesian optimization. Crystal lattice energies and densities are computed for α-, β-, and γ-glycine, and the mechanical stability of these polymorphs is tested over a range of temperatures. Solution densities and concentration-dependent self-diffusion coefficients are calculated in conjunction with three water models. Using alchemical hydration free energy calculations, we also obtain solvation and solution enthalpies. Optimization of the nonbonded parameters in OPLS force field variants improves the prediction of crystal properties for all polymorphs simultaneously. We present a force field that correctly reproduces the relative polymorph stability, remains mechanically stable beyond ambient temperatures, and gives excellent agreement with experimental lattice energies, crystal densities, and solution enthalpies. This optimized model is expected to provide accurate insight into the mechanisms controlling glycine polymorphism in complex environments. Moreover, the optimization framework developed here provides a general approach for improving force fields of other molecular crystals.

## Graphical abstract

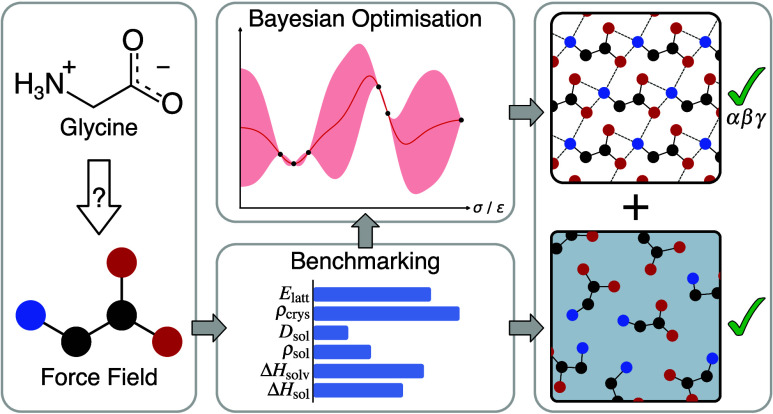

## 1 Introduction

Crystal polymorphs can exhibit significantly different physical characteristics, such as density, melting point, conductivity and optical behavior. Therefore, the ability to control polymorphic outcome is vital across many industries. This is especially true in the pharmaceutical sector, as polymorphism can affect the stability, toxicity, and efficacy of drug compounds.^1–3^ For many target molecules, mechanisms of nucleation and growth are not well understood. Computer simulations can provide molecular-level insights not available through experimental investigations.

Glycine, despite being the simplest amino acid, exhibits complex polymorphism and serves as an archetypal system for understanding polymorphic control in organic and pharmaceutical solids. It has three possible forms at ambient pressure: α-, β- and γ-glycine, shown in Figure 1. Molecules exist as zwitterions in these crystal structures and also in solution. α-glycine and β-glycine have monoclinic structures with space groups *P*2_1_/*n* (14) and *P*2_1_ (4), respectively, and exhibit hydrogen-bonded layers that differ only in their stacking. The γ-glycine polymorph has trigonal space group *P*3_1_ (144). Its helical chains of zwitterionic glycine give rise to a large cell dipole moment.^4^ Solution enthalpy measurements indicate a stability ordering of γ > α > β.^5^ However, polymorph energies are closely spaced, with enthalpy differences of Δ*H*_sol_(γ) – Δ*H*_sol_(α) = 0.27 ± 0.11 kJ mol^–1^ and Δ*H*_sol_(γ) – Δ*H*_sol_(β) = 0.59 ± 0.11 kJ mol^–1^.^5^ The free energy difference between γ- and α-glycine, inferred from heat capacity measurements, is 0.16 ± 0.15 kJ mol^–1^ at ambient temperature.^6,7^ Although γ-glycine is the most stable polymorph, it can be difficult to crystallize under ambient conditions. α-glycine grows around 500 times more quickly in aqueous solution and is typically the primary crystallization product.^8^

**Figure 1:**
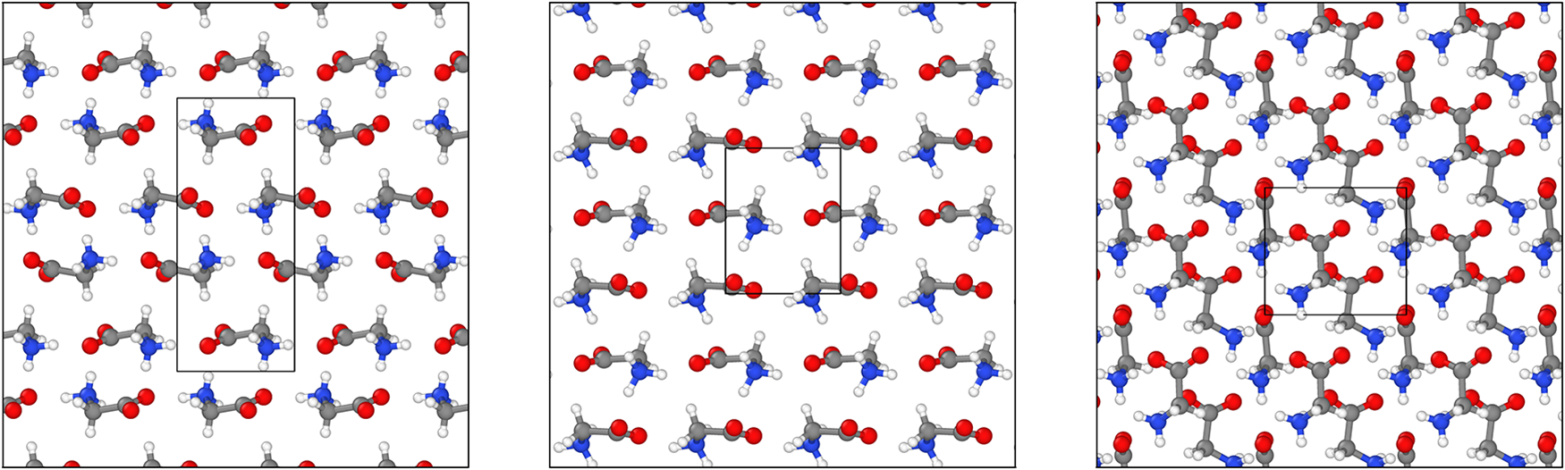
Crystal structures of the three ambient-pressure glycine polymorphs: α-glycine (left), β-glycine (center), γ-glycine (right). Inner boxes show unit cells, containing 4, 2, and 3 formula units, respectively.

This strong preference to crystallize in the α form means glycine provides a good test of polymorphic control. Many experimental techniques have been developed to achieve preferential growth of a particular polymorph. β-glycine can be precipitated through rapid addition of ethanol antisolvent.^9^ Growth of γ-glycine is favored in the presence of an electric field^10^ and in strongly acidic or basic environments.^11^ Microemulsions, ionic surfactants and process conditions have also been shown to influence glycine polymorphic outcomes.^12–14^ Moreover, crystallization in structured ternary fluid systems can allow selective growth of all three polymorphs, controlled by changes in glycine supersaturation and mixture composition.^15^

Understanding mechanisms that lead to the preferential nucleation and growth of specific polymorphs in these cases can be aided by computer simulation. Although *ab initio* methods can provide accurate calculations of various crystal properties,^16–19^ they are computationally expensive. The time scales and length scales required to investigate crystallization from solution are currently only available using classical force fields. For meaningful insights into glycine crystallization phenomena, it is vital to use a force field that not only reproduces solution properties well, but also crystal properties for all three ambient pressure polymorphs.

Many previous simulations of glycine have focused solely on solution properties, such as diffusion, clustering, and the prevalence of hydrogen-bonded dimers.^20–23^ Additional work has extended this analysis to the glycine-solution interface,^24,25^ and has examined crystallization processes more directly through studies of nanocrystal dissolution kinetics and solubilities,^26,27^ as well as heterogeneous nucleation mechanisms.^28,29^ Several studies utilize the combination of GAFF force field and CNDO charges recommended by Cheong and Boon.^25^ In their work assessing glycine force fields, this model demonstrated good performance in reproducing solution properties with the SPC/E water model.^30^ Additionally, it was the only tested force field to correctly predict a positive solution enthalpy. However, crystal properties are reproduced poorly. The force field was only validated for α-glycine and lattice energy values for this polymorph are significantly underpredicted. This poor agreement with experiment likely arises from methodological limitations in the calculation of crystal properties, which also influence the derived solution enthalpy.

Here, we build on previous efforts to obtain an accurate glycine force field, applying methodological improvements to validate the model for all three ambient pressure polymorphs. Ten variants of the OPLS force field and eight variants of the GAFF force field are compared in their ability to reproduce glycine solution and crystal properties. Included among these variants are force fields previously tested by Cheong and Boon,^25^ in addition to force fields with updated nonbonded parameters and charge-determination methods. In extending our analysis to include β- and γ-glycine, we employ an Ewald summation to ensure proper convergence of long-range electrostatics in lattice energy calculations. Moreover, we perform these calculations using fully relaxed crystal structures for which unit cell parameters are allowed to vary from their experimental values. Solution density, enthalpy, and diffusion coefficients are tested in combination with TIP3P, TIP4P and TIP4P/2005 water models.^31,32^ The mechanical stability of the crystal polymorphs is also tested across a range of temperatures. Finally, nonbonded parameters of the best-performing force fields are optimized using a multiobjective Bayesian approach to further improve agreement with experimental values and produce a model that will enable molecular insight into crystallization control.

The rest of this article is organized as follows. Section 2 details the force field variants tested in this work and describes simulation protocols used to calculate various glycine properties. We also provide an overview of the Bayesian optimization scheme used to recalibrate model parameters. In Section 3, we first assess the performance and mechanical stability of the models when reproducing crystal-phase properties, with discussion of the methodological improvements applied to these calculations. Next, we examine solution-phase behavior, analyzing densities and diffusion coefficients across different glycine concentrations, in addition to solvation and solution enthalpies. Then, we present results for our optimized glycine force field and compare its performance against the unoptimized models. Finally, Section 4 summarizes the key findings of this work, identifies the most accurate unoptimized models and discusses the applicability of our optimized model.

## 2 Methodology

### 2.1 General Protocols

Molecular dynamics simulations were performed with the GROMACS 2022.3 software package,^33,34^ using periodic boundary conditions and the leapfrog algorithm^35^ to integrate equations of motion. A cutoff of 10 Å was used to truncate Lennard-Jones (LJ) interactions and short-range electrostatic contributions. For long-range electrostatics, the particle-mesh Ewald (PME) algorithm^36^ was used with order 4, a Fourier grid-spacing of 0.12 nm, and a relative error in the forces of 10^–6^.

For simulations of glycine in solution, a correction was applied to account for dispersion interactions beyond the cutoff.^37^ Additionally, the Linear Constraint Solver (LINCS) algorithm was employed to constrain bonds including hydrogen atoms, allowing for a simulation time step of 2 fs. Simulations in the *NVT* ensemble were performed using the canonical velocity-rescaling thermostat of Bussi et al.,^38^ with a coupling time of 1 ps. Pressure coupling was added for *NpT* simulations using a stochastic cell rescaling algorithm,^39^ with a 5 ps coupling time.

Simulations of crystalline glycine were run using double precision with a simulation time step of 1 fs, treating all bonds explicitly with no constraints. Unit cells for α-, β- and γ-glycine were obtained from the Cambridge Structural Database (CSD), with refcodes GLYCIN98,^40^ GLYCIN71,^41^ and GLYCIN33,^42^ respectively. These unit cells were replicated 6 × 2 × 6, 6 × 4 × 6, and 4 × 4 × 6 times, respectively, in three dimensions to create similarly sized supercells, each with 288 molecules and a minimum cell length of 23.5 Å.

Specific simulation settings for calculation of different properties are detailed in the following sections.

### 2.2 Force Fields

The OPLS^31,43^ and GAFF^44^ force fields considered here were parametrized to fit experimental properties of small organic molecules, making them suitable for simulation of glycine. Each force field defines a collection of bonded and nonbonded parameters, generally specified for distinct atom types, which together construct an overall potential energy function for the system. Force fields often have a recommended set of point charges, but are commonly paired with other atomic charges derived through a variety of methods. Owing to variations in parameter sources, charge sets, force field versions, and some ambiguity in assigning atom types for glycine, we consider 18 different force field variants in this work. A summary is given in Table 1.

**Table 1:** Summary of Force Field Variants, Showing Sources of Force Field Parameters and Atomic Charge Sets

force field	parameters	charge set
opls-aa	OPLS^a^	default OPLS^a^
opls-chpg	OPLS^a^	6–31G*CHELPG^a^
opls-cm1a	OPLS^a^	1.14*CM1A-LBCC^45^
opls-cndo	OPLS^a^	CNDO^4^
opls2-aa	OPLS/2020 ^46^	default OPLS^a^
opls2-chpg	OPLS/2020 ^46^	6–31G*CHELPG^a^
opls2-cm1a	OPLS/2020 ^46^	1.14*CM1A-LBCC^45^
opls2-cndo	OPLS/2020 ^46^	CNDO^4^
opls3-chpg	LigParGen^47^	6–31G*CHELPG^a^
opls3-cm1a	LigParGen^47^	1.14*CM1A-LBCC^45^
gaff-bcc	GAFF^48^	AM1-BCC^49^
gaff-cndo	GAFF^48^	CNDO^4^
gaff-dnp	GAFF^48^	DNP^24^
gaff-resp	GAFF^48^	RESP^50^
gaff2-abcg	GAFF2 ^48^	ABCG2 ^51^
gaff2-cndo	GAFF2 ^48^	CNDO^4^
gaff2-dnp	GAFF2 ^48^	DNP^24^
gaff2-resp	GAFF2 ^48^	RESP^50^

a Parameters obtained from GROMACS topology files.

Parameters for the GAFF and the updated GAFF2 force fields were obtained using AmberTools24 ^48^ and paired with point charges obtained using recommended charge methods AM1-BCC^49^ and ABCG2,^51^ respectively. We also tested RESP charges generated from *ab initio* optimization of a glycine molecule at the HF/6–31G* level, with an implicit water solvent employed to stabilize the zwitterionic form.^50^ OPLS parameters were obtained from topology files included in the GROMACS software package. In addition to the basic OPLS atom types, these files contained atom types for a glycine zwitterion, with identical nonbonded parameters and partial charges derived from *ab initio* calculations according to the CHELPG scheme.^52^ The OPLS/2020 ^46^ force field is an updated version of OPLS, with improvements to many nonbonded and dihedral parameters. The LigParGen software^47^ was used to produce another OPLS variant with 1.14*CM1A-LBCC^45^ partial charges. This model differs slightly from the OPLS model in its angle parameters and LJ parameters for the carbonyl carbon (C). The CNDO^4^ and DNP^24^ charge sets tested by Cheong and Boon^25^ were also included for comparison. Point charges and nonbonded parameters for each force field variant are given in Section 1 of the Supporting Information.

Although it is recommended to use the 3-site TIP3P water model^31^ with GAFF and the 4-site TIP4P water model^31^ with OPLS, we tested each force field with both models in addition to the updated TIP4P/2005 model,^32^ keeping the glycine parameters unchanged in each case.

### 2.3 Crystal Properties

The lattice energy of each glycine polymorph was calculated using

1 $$E_{\mathrm{latt}}=\frac{E_{\mathrm{total}}}{N}-E_{\mathrm{single}}$$

where *E*_total_ is the total potential energy of the fully relaxed crystal supercell containing *N* glycine zwitterions. *E*_single_ is the potential energy of a single glycine molecule in a vacuum averaged over thermal fluctuations, where we take the same zwitterionic form as in the crystal phase to maintain a consistent bonding topology. Lattice energies cannot be measured experimentally. Instead they are inferred from experimental sublimation enthalpy values by

2 $$\Delta H_{\mathrm{sub}}=-E_{\mathrm{latt}}-2RT+\Delta E_{\mathrm{pt}}$$

where Δ*E*_pt_ is the energy change due to proton transfer as the molecule becomes zwitterionic, correcting for the gas-phase reference used in eq 1. The 2*RT* terms arises from the difference between the translational and rotational contributions to the ideal gas enthalpy (4*RT*) and the corresponding lattice vibrational and librational contributions to the crystal enthalpy (6*RT*).^53^

We obtain relaxed crystal supercells in GROMACS with a three-step procedure. First, atomic positions are relaxed using steepest-descent minimization to a maximum force tolerance of 0.1 kJ mol^–1^ nm^–1^. Then we propagate dynamics, applying anisotropic pressure coupling to allow cell parameters to relax. Finally, the potential energy is minimized with respect to atomic coordinates once more, with the cell dimensions fixed at their average value.

GROMACS is yet to implement an anisotropic version of the stochastic cell rescaling barostat,^39^ therefore the intermediate simulation is performed using the Berendsen barostat,^54^ applied for each step of the dynamics with a 5.0 ps coupling time. Although the Berendsen barostat produces incorrect volume fluctuations, its first-order coupling allows efficient exponential relaxation to yield statistically correct average supercell dimensions. Components of the barostat compressibility tensor were selected to preserve the symmetry of each supercell. Cell lengths (*a*, *b*, *c*) were allowed to vary independently, while particular angles were constrained for the monoclinic (α = γ = 90°) and trigonal (α = β = 90°, γ = 120°) supercells.

For efficiency, GROMACS will modify the triclinic simulation box during dynamics to prevent the cell from becoming excessively skewed, adding and subtracting box vectors to obtain an equivalent representation. During analysis, we undo these adjustments to ensure average cell dimensions are computed correctly. Details of this procedure are given in Section 2 of the Supporting Information.

The crystal potential energy is computed employing the PME algorithm^36^ to ensure convergence of long-range electrostatics. This is important for β- and γ-glycine, which have nonzero cell dipole moments. With periodic boundary conditions and an effectively infinite crystal supercell, we assume tinfoil boundary conditions and do not apply corrections to account for the cell dipole moment.^55,56^

The average potential energy, *E*_single_, of a single zwitterion in a vacuum was calculated from a 7.5 ns simulation at 298.15 K, using Langevin dynamics to ensure sufficient sampling of conformations. Linear and angular center of mass motion were removed every time step as a precaution against poor equipartition of kinetic energy,^57^ although this should not be a problem with stochastic dynamics.^58^ Uncertainty in the potential energy value was quantified using a block averaging procedure.^59^

To investigate the mechanical stability of crystal supercells, we also performed simulations in the *NpT* ensemble for various temperatures up to 400 K. Starting from fully relaxed configurations, supercells were equilibrated for 5 ns using the Berendsen barostat.^54^ During this equilibration, the temperature was increased from 50 K up to a target temperature over 4 ns and held for a further 1 ns. Production simulations employed the Parrinello–Rahman^60^ barostat, with a 10 ps coupling time, for a further 5 ns of simulation at fixed temperature.

### 2.4 Solution Properties

Mixtures of glycine and water were prepared for 8 different mole fractions *x*_gly_, described in Table 2, by randomly inserting the appropriate number of glycine molecules into a cubic box containing 2000 water molecules. Equilibrated configurations, with box lengths of approximately 40 Å, were simulated in the *NpT* ensemble for 10 ns with isotropic pressure coupling. Solution density, ρ_sol_, was computed every 2 ps. An automated block averaging procedure was used to calculate the average density and estimate its uncertainty.^59^

**Table 2:** Compositions of Glycine in Water

*N*_gly_	*N*_wat_	*x*_gly_
20	2000	0.01
40	2000	0.02
61	2000	0.03
83	2000	0.04
105	2000	0.05
127	2000	0.06
150	2000	0.07
173	2000	0.08

An additional 10 ns of *NVT* simulation was performed for each mixture, writing particle coordinates every 2 ps. A custom analysis class, written using MDAnalysis,^61^ was used to compute the mean squared displacement (MSD) ⟨Δ**r** ^2^(τ)⟩ of the center of mass of each glycine molecule over an interval τ. Diffusion coefficients were calculated using the Einstein relation

3 $$D=\frac{1}{6}\lim_{\tau \to \infty }\frac{d}{d\tau }\left\langle \Delta r^{2}\left(\tau \right)\right\rangle$$

by fitting MSD data up to τ = 2 ns, corresponding to the range where the slope of ln⟨Δ**r** ^2^(τ) ⟩ against ln­(τ) is approximately 1.

### 2.5 Solution Enthalpy

The solution enthalpy of each crystal was computed using

4 $$\Delta H_{\mathrm{sol}}=\Delta H_{\mathrm{solv}}-E_{\mathrm{latt}}$$

A positive value indicates that crystal growth is an exothermic process. The solvation enthalpy, Δ*H*_solv_, can be obtained from the integrated Gibbs–Helmholtz relation

5 $$\frac{\Delta G_{2}}{T_{2}}-\frac{\Delta G_{1}}{T_{1}}=\Delta H_{\mathrm{solv}}\left(\frac{1}{T_{2}}-\frac{1}{T_{1}}\right)$$

Determination of the solvation enthalpy, therefore, requires calculation of solvation free energies at two distinct temperatures as well as the previously calculated lattice energy.

For glycine, we computed hydration free energies at 298.15 and 318.15 K using an alchemical approach in which the amino acid solute was decoupled from its water environment along a pathway parametrized by λ, ranging from 0 (glycine-water interactions present) to 1 (no solute–solvent interactions). This pathway consisted of 25 intermediate states, each constituting a separate simulation of a single glycine zwitterion solvated in a cubic box of side length 30 Å. Simulations were run for 5 ns, after energy minimization and 250 ps of equilibration in both the *NVT* and *NpT* ensembles. Pressures were maintained at 1 bar and temperatures controlled by the use of Langevin dynamics, which ensures proper sampling at λ states close to 1, where the zwitterion is almost completely decoupled from its environment. Coulomb interactions between solute and solvent were turned off linearly, over 10 states, according to

$$U_{\mathrm{ij}}^{\mathrm{coul}}\left(r_{\mathrm{ij}};\lambda \right)=\left(1-\lambda \right)\frac{e^{2}}{4\pi \varepsilon_{0}}\frac{q_{i}q_{j}}{r_{\mathrm{ij}}}$$ 6

The Beutler soft-core potential,^62^

7 $$U_{\mathrm{ij}}^{\mathrm{LJ}}\left(r_{\mathrm{ij}};\lambda \right)=4\epsilon_{\mathrm{ij}}\left(1-\lambda \right)\left[\frac{1}{{\left(\alpha \lambda +{\left(r_{\mathrm{ij}}/\sigma_{\mathrm{ij}}\right)}^{6}\right)}^{2}}-\frac{1}{\left(\alpha \lambda +{\left(r_{\mathrm{ij}}/\sigma_{\mathrm{ij}}\right)}^{6}\right)}\right]$$

with α = 0.5, was used to decouple LJ interactions over 15 intermediate states. Glycine intramolecular interactions were present across the whole thermodynamic pathway. Potential energies for each alchemical state were evaluated every 0.2 ps. The central carbon of the glycine zwitterion was fixed at the center of the simulation cell to improve reproducibility of free energy estimates.

Hydration free energy values were evaluated with the alchemlyb Python package,^63^ employing the MBAR estimator^64^ and built-in routines for equilibrium detection and decorrelation of potential energy data. Uncertainties in the free energy values were estimated using a bootstrapping approach with 100 samples.

### 2.6 Bayesian Optimization

Multiobjective Bayesian optimization was used to calibrate the LJ parameters of the best-performing force fields, in order to better reproduce crystal properties for the three glycine polymorphs. This approach is well-suited to the optimization of functions for which gradients cannot be computed (black-box functions), evaluation of the objective function is costly, and the dimensionality of input parameters is low. The optimization was performed using the Adaptive Experimentation Platform^65^ and the underlying BoTorch Python package.^66^

The model input $x_{\mathrm{LJ}}\in X$ was 10-dimensional, as we optimized two LJ parameters (σ and ϵ) for five distinct glycine atom types. These parameters govern both intermolecular and intramolecular nonbonded interactions, and the search space X was limited to keep them physically meaningful. σ and ϵ values were constrained to within ± 25% and ± 50% of their original force field values, respectively.

At each optimization iteration, lattice energies and unit cell parameters were calculated as described previously and used to evaluate a set of six objective functions

8 $$f\left(x_{\mathrm{LJ}}\right)=\left(f_{\mathrm{elatt}}^{\alpha },f_{\mathrm{elatt}}^{\beta },f_{\mathrm{elatt}}^{\gamma },f_{\mathrm{cell}}^{\alpha },f_{\mathrm{cell}}^{\beta },f_{\mathrm{cell}}^{\gamma }\right)$$

that aimed to improve reproduction of experimental lattice energies and unit cell parameters for all three glycine polymorphs. Each objective function took the general form of the root-mean-square fractional error

9 $$f^{p}\left(x_{\mathrm{LJ}}\right)=\sqrt{\frac{1}{d}\sum_{i}^{d}{\left(\frac{y_{i}^{p}-y_{\exp,i}^{p}}{y_{\exp,i}^{p}}\right)}^{2}}$$

of a set of properties **y** ^*p*^ calculated for a given polymorph *p* with respect to experimental values **y**_exp_^*p*^. For *f*_cell_^*p*^, **y** = (*a*, *b*, *c*, α, β, γ) and *d* = 6. For *f*_elatt_^*p*^, **y** = (*E*_latt_) and *d* = 1 so the objective function reduces to the absolute fractional error in the lattice energy

10 $$f_{\mathrm{elatt}}^{p}\left(x_{\mathrm{LJ}}\right)=\left|\frac{y_{i}^{p}-y_{\exp,i}^{p}}{y_{\exp,i}^{p}}\right|$$

Each objective function was modeled independently using a Gaussian process (GP) surrogate model

11 $$f\left(x_{\mathrm{LJ}}\right)∼\mathrm{GP}\left(m\left(x_{\mathrm{LJ}}\right),k\left(x_{\mathrm{LJ}},x_{\mathrm{LJ}}^{\prime }\right)\right)$$

which provided a probabilistic approximation of the true objective function, based on previously evaluated points, and was used to guide selection of new sampling points during optimization. We used a constant mean function *m*(**x**_LJ_) = μ and radial basis function (RBF) kernel

12 $$k\left(x_{\mathrm{LJ}},x_{\mathrm{LJ}}^{\prime }\right)=\exp\left(-\frac{∥x_{\mathrm{LJ}}-x_{\mathrm{LJ}}^{\prime }{∥}^{2}}{2l^{2}}\right)$$

where the kernel length scale *l* was optimized during model training. The model was initialized with 15 quasi-random points, generated with a Sobol sequence.

Objective function evaluations were added to the data set of sampled points $D_{n}$ and used to update the GP model posterior distributions. Subsequent sampling points were selected using

13 $$x_{\mathrm{LJ}}^{n+1}=\underset{x_{\mathrm{LJ}}\in X}{\arg\;\max\;}\alpha \left(x_{\mathrm{LJ}}^{n};D_{n}\right)$$

with acquisition function α. For this multiobjective problem, we used the quasi-Expected Hypervolume Improvement (qEHVI) acquisition function, which aims to expand the Pareto front (the set of solutions where improvement in one objective worsens another) given by the GP models. The optimization process was repeated up to a maximum of 100 iterations. Using this technique, we aim to efficiently sample the parameter space X , balancing exploration of regions where GP surrogate model uncertainty is high and exploitation of parameter sets **x**_LJ_ predicted to yield low objective values.

## 3 Results and Discussion

### 3.1 Crystal Properties

Lattice energies and crystal densities of the relaxed, energy-minimized polymorph supercells are shown in Figure 2. Using eq 2, with an experimental sublimation enthalpy value of 136.4 ± 0.4 kJ mol^–1^, measured for an unspecified glycine polymorph at 455 K,^67^ and a DFT-calculated proton transfer energy of −138.2 kJ mol^–1^,^24^ we obtain a reference lattice energy of −282.17 kJ mol^–1^. Accounting for the factor of 2 difference in the definition of *E*_latt_ for Cheong and Boon^25^ and separating out Δ*E*_pt_ from the value given by Xavier et al.,^19^ we recover similar reference values of −279 and −282.2 kJ mol^–1^, respectively. Solution enthalpy measurements indicate a polymorph stability ordering of γ > α > β, with all three lattice energies falling within a 1.0 kJ mol^–1^ range.^5^ Therefore, the plots show a single experimental line for all three polymorphs.

**Figure 2:**
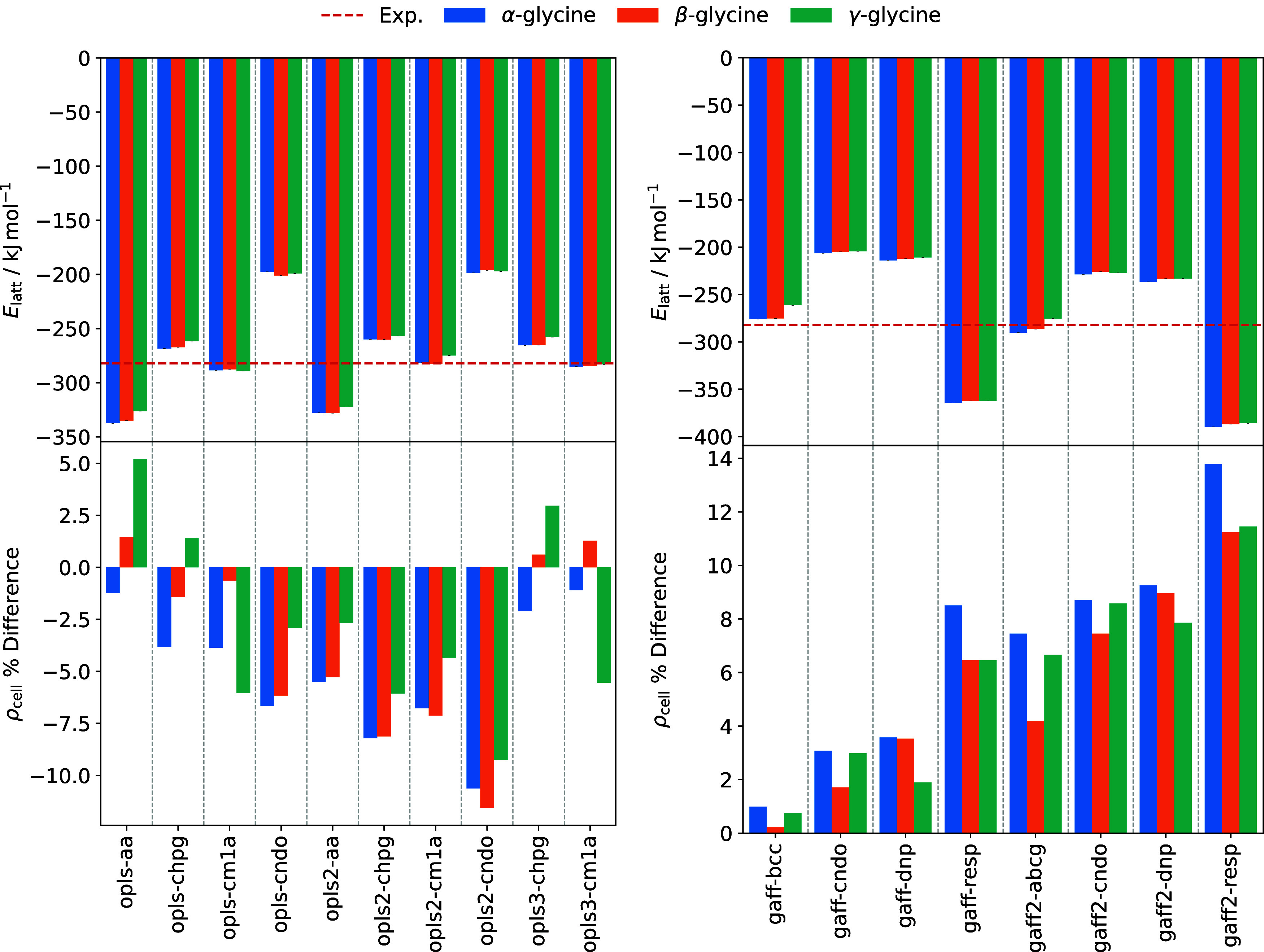
Crystal properties of glycine polymorphs for OPLS (left) and GAFF (right) force fields variants. The lattice energy *E*_latt_ reference value (red dashed line) is derived from the experimental sublimation enthalpy.^67^ Cell density ρ_cell_ is given as a percentage difference from 0 K reference values obtained through a fit of crystallographic measurements at various temperatures.^42^

Generally, force fields utilizing the same charge set show similar lattice energy values, indicating that this property is largely determined by electrostatic contributions. Variants opls-cm1a, opls2-cm1a and opls3-cm1a give the best agreement with experiment, predicting lattice energies of all three polymorphs to within 2.6%. OPLS force fields utilizing CHELPG charges (opls-chpg, opls2-chpg and opls3-chpg) also perform reasonably well, but underpredict lattice energy magnitudes by up to 9.0%. For the GAFF force fields, gaff2-abcg and gaff-bcc give the best performance, with maximum errors of 2.9 and 7.4%, respectively. Although lattice energies are largely electrostatic in nature, the LJ parameters of each force field can influence the relative stability of polymorphs. Almost all force fields predict an incorrect stability ordering, with most underestimating the relative stability of the γ form. Only opls-cm1a predicts the correct order of γ > α > β.

Lattice energy deviations are generally much smaller than those reported previously for α-glycine.^25^ However, similar trends appear: models using the CNDO and DNP charge sets significantly underestimate the magnitude of the lattice energy. Here, the gaff2-resp (+38.1%), opls2-cndo (−30.5%) and gaff-cndo (−27.6%) variants give the largest deviations from experiment. Absolute lattice energy values can diverge significantly from earlier reports. For example, we expect a value of −153 kJ mol^–1^ for the gaff-dnp force field to be consistent with previous studies,^24,25^ but we obtain −213.9 kJ mol^–1^. This difference may be attributed to various factors. First, although point charges are given explicitly, exact details of each force field are not reported in all cases, so the parameters used here may be inconsistent. Disagreement may also arise from differences in calculation methods. Cheong and Boon^25^ compute the lattice energy with a direct summation over intermolecular interactions, following energy minimization of the crystal supercell. Although convergence may be achieved for centrosymmetric α-glycine (which has no cell dipole moment) with a large supercell, the same method cannot be applied for the β and γ forms considered here. We instead compute the lattice energy, using eq 1, by employing the PME algorithm^36^ to ensure convergence of long-range electrostatic interactions. Computing the lattice energy in this way also accounts for conformational differences between glycine in the gas-phase and the crystal-phase. Geometries of glycine monomers in the crystal form are compared to experiment in Section 3 of the Supporting Information.

Additionally, calculations performed by Cheong and Boon^25^ utilize an α-glycine supercell constructed from a different CSD structure, measured at higher temperature.^68^ They constrain supercell lengths and angles at these experimental values during their calculations, while we allow these parameters to vary to give a fully relaxed structure. Table 3 shows that allowing these parameters to relax can stabilize structures such that the magnitude of the lattice energy is increased by up to 20.77 kJ mol^–1^. In many cases, this is a significant fraction of the lattice energy, and sufficient to alter the relative stability of polymorphs. Calculating the lattice energies in this way is preferable, as it provides a self-consistent measure of the lattice energy based on the model’s own optimized geometry, independent of any experimental structure.

**Table 3:** Reduction in Crystal Lattice Energy, Given in kJ mol^–1^, after Allowing Unit Cell Parameters to Relax during Energy Minimization

force field	α-glycine	β-glycine	γ-glycine
opls-aa	7.65	11.41	5.39
opls-chpg	6.14	6.61	1.31
opls-cm1a	9.07	9.05	16.38
opls-cndo	5.75	4.31	0.24
opls2-aa	3.48	3.12	0.14
opls2-chpg	6.33	3.77	1.97
opls2-cm1a	5.94	5.54	1.35
opls2-cndo	14.63	8.06	7.34
opls3-chpg	3.81	4.56	2.11
opls3-cm1a	6.48	9.05	15.20
gaff-bcc	2.22	5.12	4.77
gaff-cndo	1.95	2.37	3.25
gaff-dnp	2.55	3.28	3.81
gaff-resp	4.02	5.94	17.56
gaff2-abcg	5.40	3.98	7.20
gaff2-cndo	4.86	5.46	6.80
gaff2-dnp	5.55	6.19	6.58
gaff2-resp	9.87	13.59	20.77

Allowing relaxation of lattice parameters also means that we can assess how well each force field reproduces experimental lattice densities at 0 K. These results are shown in Figure 2, and unit cell parameters for key force field variants are tabulated in Section 4 of the Supporting Information. There are few low-temperature entries in the CSD for β- and γ-glycine.^69^ Therefore, results are given as percentage differences from values obtained from a fit of crystallographic measurements at various temperatures.^42^ Details of this fitting procedure are given in Section 5 of the Supporting Information.

Lattice densities show a weak dependence on charge set and are instead determined primarily by the LJ parameters of each force field. All variants of the GAFF force field overestimate the density of the glycine polymorphs. The gaff-bcc force field shows excellent agreement with experiment, predicting densities to within 1%. Performance is also reasonable for the gaff-cndo and gaff-dnp models, with maximum deviations of up to 4%. The GAFF2 force field introduces a new atom type for the ammonium nitrogen (N) with a smaller σ parameter, and the crystal densities are therefore generally much higher. Conversely, the OPLS/2020 models underpredict crystal densities due to an increased σ parameter for the ammonium nitrogen (N). The other OPLS models exhibit densities correct to within 6%.

Overall, the gaff-bcc force field performs well in modeling the crystal properties of glycine polymorphs. It gives very good predictions for crystal densities and reasonable lattice energy values, although it underestimates the stability of the γ-glycine polymorph. Deviations in the crystal densities are larger for opls-cm1a, opls2-cm1a and opls3-cm1a. However, lattice energy predictions are very good for these models, particularly for opls-cm1a which reproduces the correct order of polymorph stability.

For models that accurately reproduce properties at 0 K, we also examined the stability of crystal forms at finite temperatures (see Section 6 of the Supporting Information for details). Many models become mechanically unstable at these elevated temperatures. In particular, the gaff-bcc and gaff-cndo force fields exhibit unphysical behavior for α- and β-glycine, respectively. The opls-cm1a and opls3-cm1a variants, however, remain stable for all three polymorphs up to 400 K and reproduce the experimental temperature dependence of unit cell volumes reasonably well.

### 3.2 Solution Properties

The properties of glycine in aqueous solution depend not only on the tested force field variants but also on the underlying water model. We therefore test each force field in combination with three different water models: TIP3P, TIP4P and TIP4P/2005.^31,32^ Results for all combinations are given in Section 7 of the Supporting Information. For clarity, the figures below only show results for the OPLS force field variants in combination with TIP4P/2005 water and the GAFF models with TIP3P water. Figure 3 shows densities of glycine in aqueous solution as a function of its mole fraction. The experimentally measured solubility of glycine in water is 249.9 g L^–1^ at 298.15 K.^70^ Assuming this solubility is reproduced faithfully by our models, a mole fraction of 0.057 corresponds to a saturated solution and the maximum mole fraction of 0.08 to a supersaturation of 1.4.

**Figure 3:**
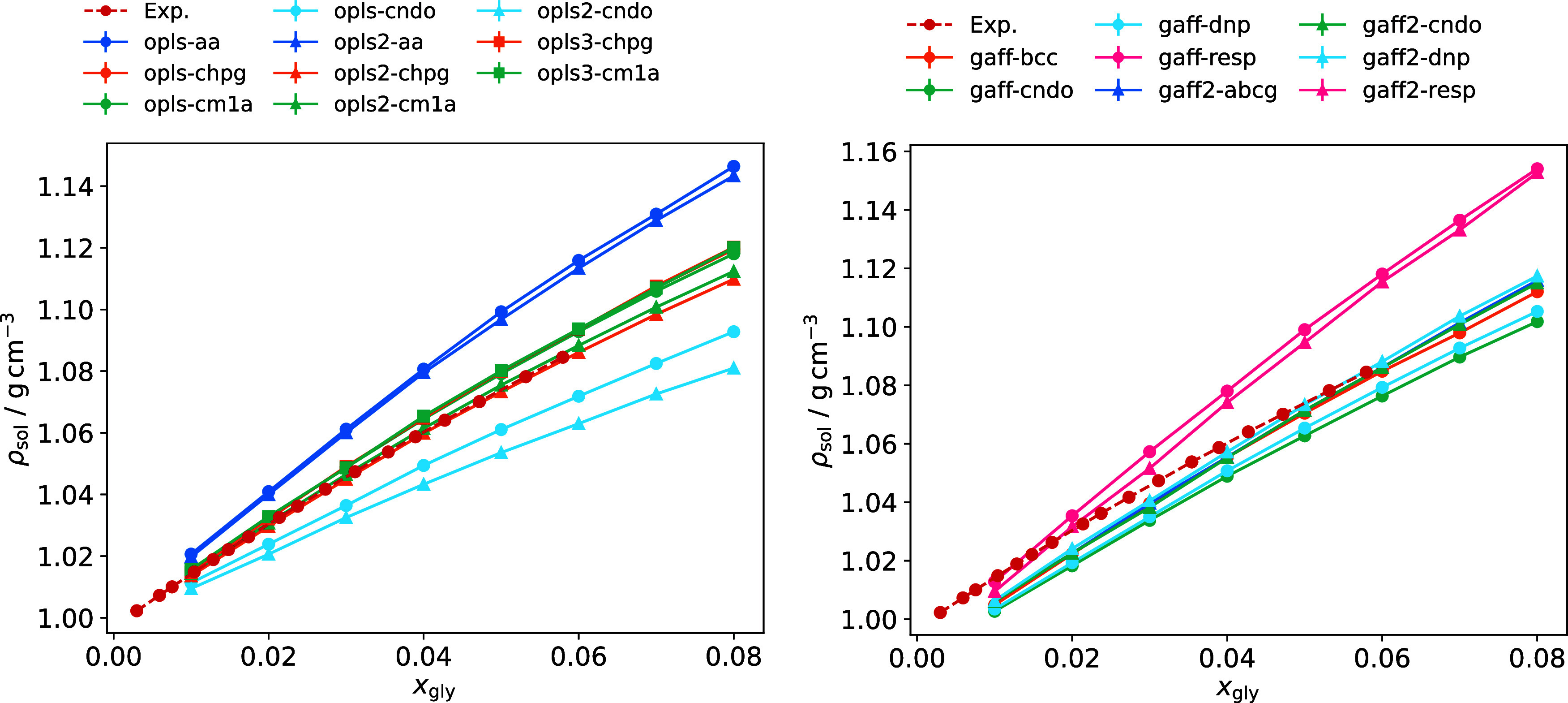
Density of glycine-water solution as a function of glycine mole fraction for OPLS force fields with TIP4P/2005 water (left) and GAFF force fields with TIP3P water (right), compared with experimental data from Dalton and Schmidt.^70^ Lines with the same color utilize the same charge set, and lines with the same marker shape have the same nonbonded parameters.

Glycine solution density depends strongly on the water model. The TIP3P model is known to underestimate the density of pure water, with a value of 0.98 g cm^–1^ at 298.15 K.^31^ 4-site water models TIP4P and TIP4P/2005 perform better, slightly overpredicting densities of 1.001 and 0.9979 g cm^–1^, respectively.^32^ This effect is shown clearly at low mole fractions; solution densities computed using the TIP3P model are all underpredicted compared to experiment. Solution densities increase for the 4-site water models, and the slope of the experimental curve is reproduced more closely.

Comparing curves for opls-cndo, opls2-cndo, gaff-cndo and gaff2-cndo, GAFF models tend to give higher solution densities, just as they predict larger densities in the crystal phase. However, density in solution shows a much greater dependence on electrostatics. Curves for models using the same point charges are very similar. While small differences in LJ parameters can influence solution density (e.g., gaff-dnp and gaff2-dnp, opls2-cm1a and opls3-cm1a), changing charge set can bring about much larger differences. The gaff-resp and gaff2-resp models overpredict the density by a large margin. According to Cheong and Boon,^25^ the gaff-cndo model together with the 3-site SPC/E water model^30^ reproduces solution properties very well. Here, this model also performs well with the 4-site TIP4P/2005 water model (Figure S6). For the OPLS force fields, opls2-cm1a and opls2-chpg also show excellent agreement with experiment in combination with TIP4P and TIP4P/2005 water. The other CM1A and CHELPG models slightly overpredict the solution density with these 4-site models.

The self-diffusivity of glycine was also investigated, with results shown in Figure 4 and representative MSD curves given in Section 8 of the Supporting Information. Glycine diffusion is expected to decrease as its mole fraction increases and intermolecular interactions become more prevalent. All force field combinations tested here show this general trend. Error bars are given by the standard error in the mean of individual glycine zwitterion diffusion coefficients. Therefore, uncertainty is reduced at higher mole fractions, where more glycine molecules are present in the simulation box.

**Figure 4:**
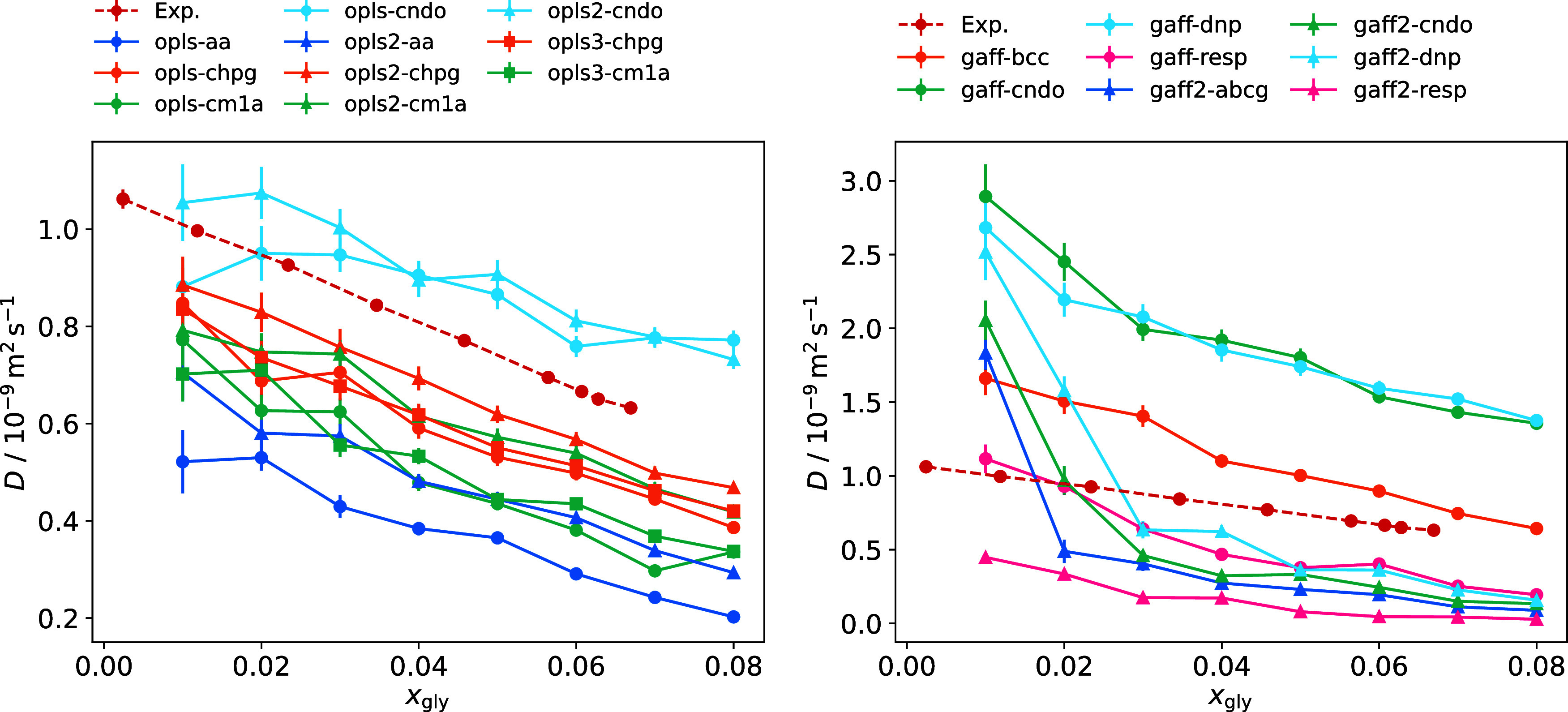
Glycine diffusion coefficient as a function of mole fraction for OPLS force fields with TIP4P/2005 water (left) and GAFF force fields with TIP3P water (right), compared with experimental data (red circles) from Huang et al.^71^ Lines with the same color utilize the same charge set, and lines with the same marker shape have the same nonbonded parameters.

The diffusion of glycine in aqueous solution depends strongly on the intrinsic mobility of water, which can vary significantly between different models. The TIP3P water model overestimates the self-diffusion coefficient of pure water, with a value of 5.39 × 10^–9^ m^2^ s^–1^ compared to the experimental value of 2.27 × 10^–9^ m^2^ s^–1^.^25^ Accordingly, most OPLS force fields in combination with this model overestimate the diffusivity of glycine (Figure S7). TIP4P and TIP4P/2005 improve on this prediction, with values of 3.9 × 10^–9^ m^2^ s^–1^ and 2.08 × 10^–9^ m^2^ s^–1^, respectively.^32^ Diffusion curves shift to lower values for these 4-site water models, reducing variability between different glycine force fields. Many OPLS models give diffusion values that lie close to experiment when combined with the TIP4P model. However, the slope of the experimental curve is better reproduced by the TIP4P/2005 model, particularly at low mole fractions.

Diffusion coefficients are known to be sensitive to small changes in force field parameters, and this sensitivity may be further amplified for zwitterionic species, such as glycine, where strong electrostatic interactions play a dominant role. Consequently, significant variability in diffusion curves is observed, even for a given water model, consistent with trends reported for glycine and other small molecules in previous simulation studies.^25,72^ Curves for the OPLS models tend to cluster according to their assigned partial charges. This is not the case for the GAFF force fields, indicating that nonbonded interactions play a greater role in their diffusion behavior. GAFF2 models tend to be less mobile than those using GAFF parameters.

Overall, the TIP4P/2005 water model shows the best performance in capturing the variation of solution density and glycine self-diffusivity across the range of mole fractions. For the GAFF force fields, gaff-cndo and gaff-dnp reproduce solution properties well with this model. OPLS models utilizing the CM1A and CHELPG charge sets all perform reasonably well in reproducing solution densities, although they underestimate glycine mobility.

### 3.3 Solution Enthalpy

In eq 4, the solution enthalpy Δ*H*_sol_ is decomposed into two terms: the crystal lattice energy *E*_latt_ and the solvation enthalpy Δ*H*_solv_, which describes the enthalpy change upon transfer of a molecule from vacuum into solution. The balance of these contributions determines the enthalpy change for transferring a molecule from its crystal form into solution. As Δ*H*_sol_ incorporates both solution and crystal properties, it is an informative target for force field validation. A positive value indicates an enthalpic preference for crystal growth, and is therefore desirable for a force field designed to probe glycine crystallization mechanisms. Based on simulations of the glycine crystal–solution interface, Cheong and Boon^25^ suggest that reproduction of the solution enthalpy is the primary requirement for a force field to model crystal growth from solution effectively.

Here we show results for the α-glycine polymorph. Values for the β and γ forms will differ only in their lattice energy contributions, so a force field that predicts lattice energies accurately should yield similar solution enthalpies for all three forms. The experimental solution enthalpy for α-glycine is 14.5 ± 0.8 kJ mol^–1^.^5^ Using eq 4 and our reference lattice energy of −282.17 kJ mol^–1^, we therefore expect a solvation energy of −267.7 ± 0.8 kJ mol^–1^. Figure 5 shows solvation enthalpy and solution enthalpy values for all force field and water model combinations.

**Figure 5:**
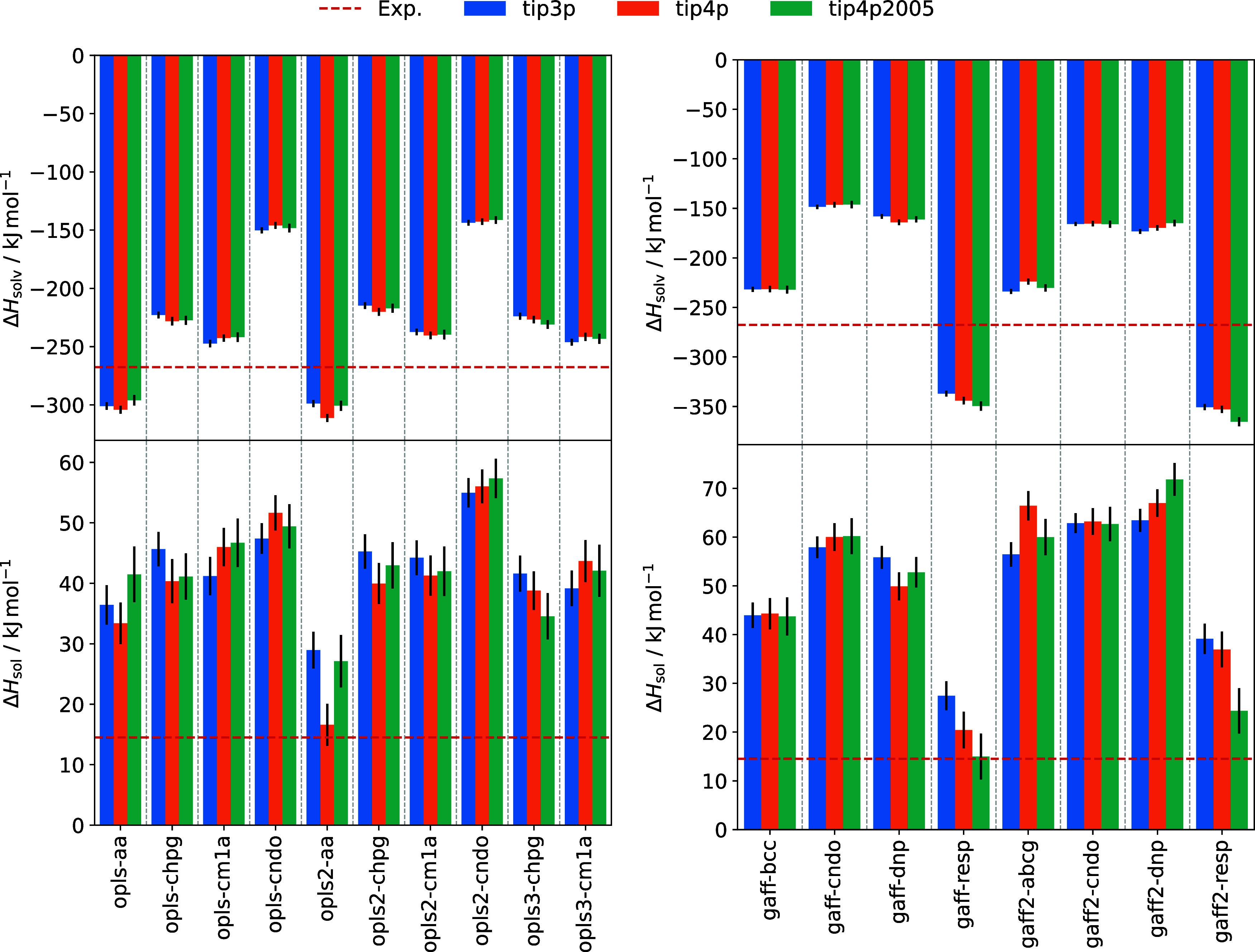
Solvation enthalpy and α-glycine solution enthalpy values for OPLS (left) and GAFF (right) force field variants in combination with different water models, compared with experimental data (red dashed lines) from Perlovich et al.^5^

Computing solvation enthalpy using eq 5 requires accurate hydration free energy estimates. We performed checks to ensure good time convergence of simulations and sufficient potential energy overlap between neighboring λ-states. Details are given in Section 9 of the Supporting Information. Solvation enthalpy values are largely independent of the water model and seem to be determined by electrostatic force field contributions, showing similar trends to lattice energies. OPLS models opls-aa and opls2-aa overpredict the magnitude of the solvation enthalpy by up to 17%, while variants utilizing CM1A charges underpredict the solvation enthalpy by around the same amount. The gaff-bcc and gaff2-abcg models, which gave good lattice energy predictions, show the best performance reproducing the experimental solvation enthalpy to within 14%. Estimates for the opls-aa, opls-cndo and gaff-cndo models are comparable to the values given by Cheong and Boon^25^ for the OPLS/default (−304.7 kJ mol^–1^), OPLS/CNDO (−149.1 kJ mol^–1^) and GAFF/CNDO (−141.9 kJ mol^–1^) force fields.

In contrast to the results of Cheong and Boon,^25^ which show only one positive value, all force fields tested in this work yield positive solution enthalpies. Therefore, we expect crystal growth to be exothermic for all tested models. This difference arises due to the methodological improvements, discussed earlier, in calculating lattice energies for the crystal polymorphs. Correctly capturing the stability of the polymorphs gives more negative lattice energies which, using eq 4, leads to larger solution enthalpy values. Accurate lattice energy values are therefore essential when using solution enthalpy as a measure of force field quality.

As both lattice energy and solvation enthalpy depend strongly on electrostatics, solution enthalpy exhibits this same dependence. The opls2-aa model, with TIP4P water, and the gaff-resp model, with TIP4P/2005 water, both reproduce the experimental solution enthalpy value within their range of error. In both cases, however, this is due to an underpredicted solvation enthalpy and an overpredicted lattice energy which combine to give a reasonable solution enthalpy. As this property is a key benchmark in assessing model performance, it is desirable to achieve agreement with experiment without these compensating errors.

### 3.4 Force Field Optimization

While many force fields tested in this work show good performance for specific properties, no single force field shows excellent agreement with experiment for all solution-phase and crystal-phase properties. The gaff-bcc model reproduces static crystal properties well, but shows mechanical instabilities at higher temperatures. The gaff-cndo and gaff-dnp force fields, which show good performance for glycine in solution, significantly underpredict lattice energy values. It is clear, however, that for properties that depend strongly on electrostatics, the CM1A charge set outperforms most others and that the TIP4P/2005 water model delivers good solution properties. On this basis, we deem the opls-cm1a, opls2-cm1a and opls3-cm1a force fields to be suitable candidates for further calibration.

These models give excellent predictions for crystal lattice energies but underpredict the solvation enthalpy of glycine, resulting in solution enthalpies that are too large. Combined with TIP4P/2005 water, they show good agreement with experimental solution densities and reproduce glycine diffusion coefficients moderately well. Though slightly better in predicting solution properties, the opls2-cm1a model underperforms in reproducing crystal densities for the glycine polymorphs. Our results indicate that crystal density is primarily influenced by nonbonded parameters. Compared to the effect of point charges, variations in these nonbonded parameters have a small impact on crystal lattice energies. Therefore, by tuning the LJ parameters we should be able to improve crystal densities without compromising excellent agreement with lattice energies.

The multiobjective Bayesian optimization approach set out in Section 2.6 was used to calibrate the nonbonded parameters for five glycine atom types. σ and ϵ parameters were allowed to vary from their original values by up to 25% and 50%, respectively. Six objective functions were used to target simultaneous improvement in predicted crystal-phase properties for all three glycine polymorphs. Additionally, a constraint was applied to bias the underlying GP surrogate models toward nonbonded parameters that give a correct polymorph stability ordering of γ > α > β.

Here, we present an optimized glycine model, with nonbonded parameters (shown in Table 4) obtained from recalibration of the opls2-cm1a force field. Figure 6 shows the effect of the constraint, biasing optimization toward LJ parameters that correctly reproduce the relative stability of polymorphs. As the number of trials increases, the surrogate models are able to map the optimization landscape more accurately and target force field parameters that give similar lattice energies for each polymorph.

**Figure 6:**
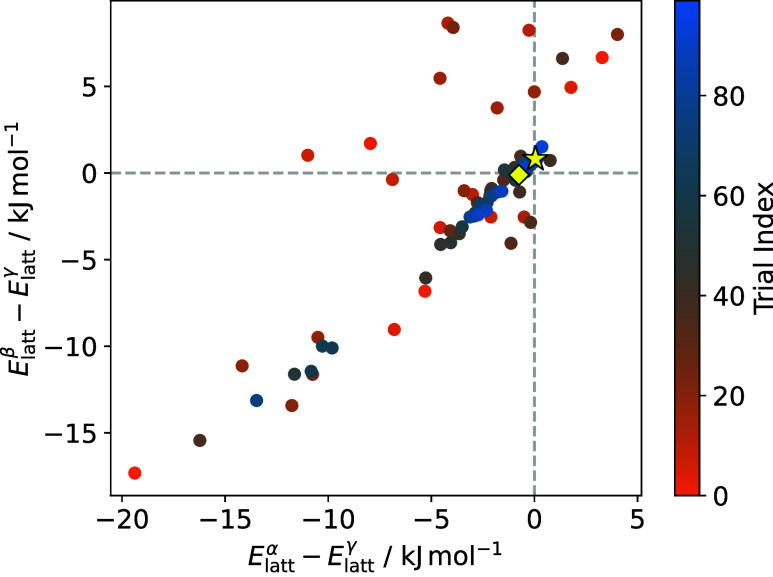
Relative lattice energies of the α-, β-, and γ-glycine polymorphs calculated for nonbonded parameter sets trialed during optimization. Trials that give the correct stability ordering appear in the upper right quadrant, close to the origin. The best-performing parameter set, selected for further analysis, is highlighted by a yellow star and the final optimization iteration by a yellow diamond.

**Table 4:** Optimized LJ Parameters for Glycine Atom Types and Their Percentage Change from Original Force Field Values

atom	σ/Å	% change	ϵ/kJ mol^–1^	% change
N	3.82094	+9.8	1.035759	–14.6
H	0.00000	0.0	0.000000	0.0
CA	4.11869	+17.3	0.270388	–2.1
HA	2.03237	–18.0	0.102125	–6.1
C	3.50422	–6.6	0.293524	–33.2
O	2.53533	–14.3	1.001103	+13.9

Crystal properties of the Bayesian optimized model are shown in Figure 7. The recalibrated force field gives lattice energy values of −282.80, −282.03, and −282.85 kJ mol^–1^ for α-, β- and γ-glycine, respectively. These values show excellent agreement with the experimental reference of −282.17 kJ mol^–1^. Moreover, relative polymorph stabilities are predicted correctly, with lattice energy differences comparable to those derived from solution enthalpy measurements. Compared to the original opls2-cm1a model, crystal densities at 0 K are improved and reproduce experimental values to within 1.3, 1.9, and 2.5%, respectively, for the α, β and γ forms. Glycine monomer geometries and unit cell parameters for the optimized model are compared to experiment in Sections 3 and 4 of the Supporting Information, respectively. This calibration of LJ parameters also improves the mechanical stability of the model when simulating crystal polymorphs at higher temperatures. Up to 350 K, the relative densities of α-, β- and γ-glycine are reproduced in the correct order and with maximum deviations of 2.5, 3.0, and 3.7%, respectively.

**Figure 7:**
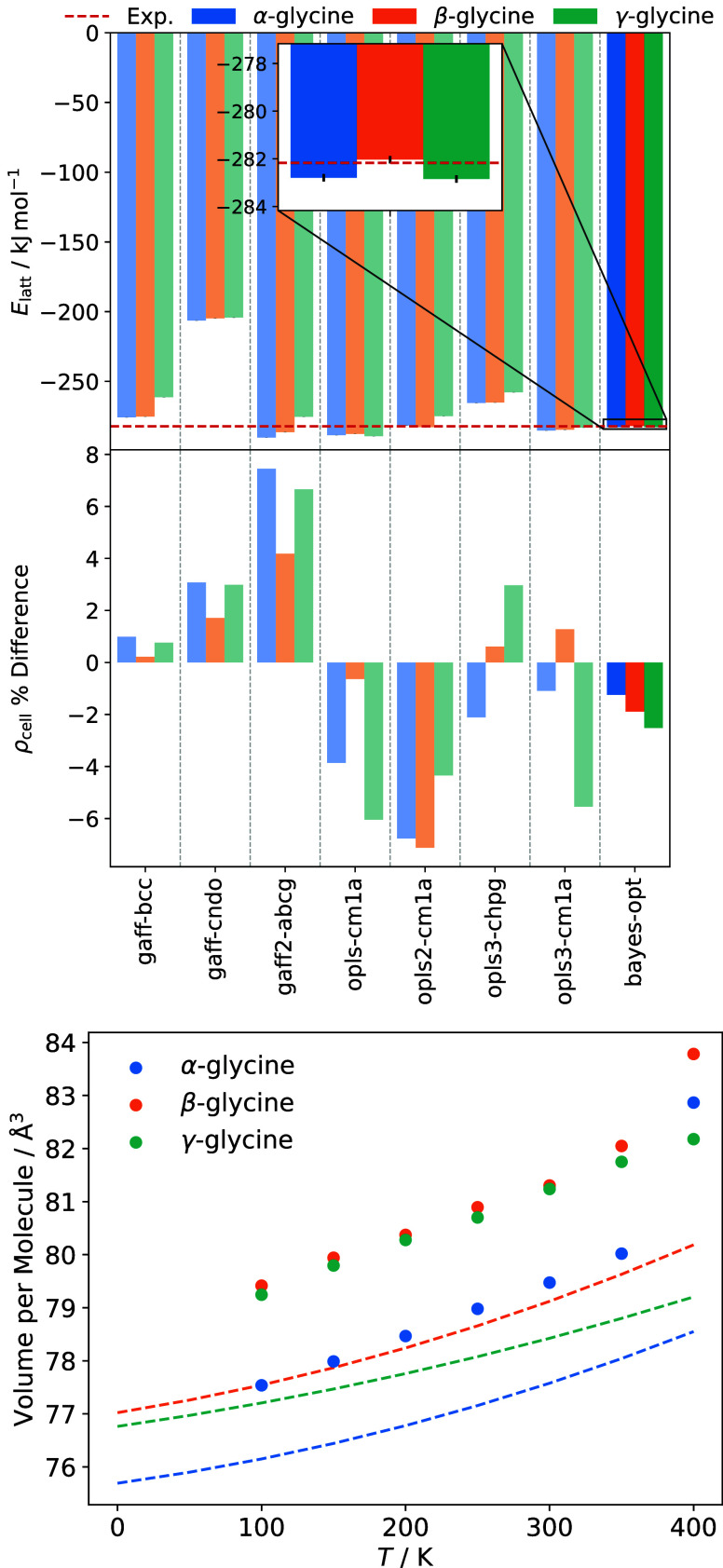
Crystal-phase properties of glycine, highlighting the performance of the Bayesian optimized model compared to previously assessed force field variants (top). The lattice energy *E*_latt_ reference value (red dashed line) is derived from the experimental sublimation enthalpy.^67^ Thermal expansion of polymorphs as predicted by the Bayesian optimized model (bottom). Dashed lines show experimental values obtained from a fit of crystallographic measurements at various temperatures.^42^

Improvements in crystal properties are balanced against a larger overprediction of glycine solution density, shown in Figure 8. The diffusion of glycine is reduced compared to the unoptimized opls2-cm1a model, showing similar performance to the opls3-cm1a curve. The predicted diffusion curve remains within a physically reasonable range of the experimental values, suggesting that optimization does not come at the cost of solution-phase dynamical accuracy. Despite calibration targeting only crystal-phase properties, the Bayesian optimized force field also shows improvement in reproducing glycine solvation enthalpy. Figure 8 shows these results. The value of −270 ± 4 kJ mol^–1^ agrees with the experimental value −267.7 ± 0.8 kJ mol^–1^ within its range of error. As discussed previously, good solution enthalpy estimates require accurate lattice energy calculations. With well-predicted polymorph lattice energies and quantitative reproduction of the experimental solvation enthalpy, the model exhibits excellent agreement with the experimental solution enthalpy, without compensating errors.

**Figure 8:**
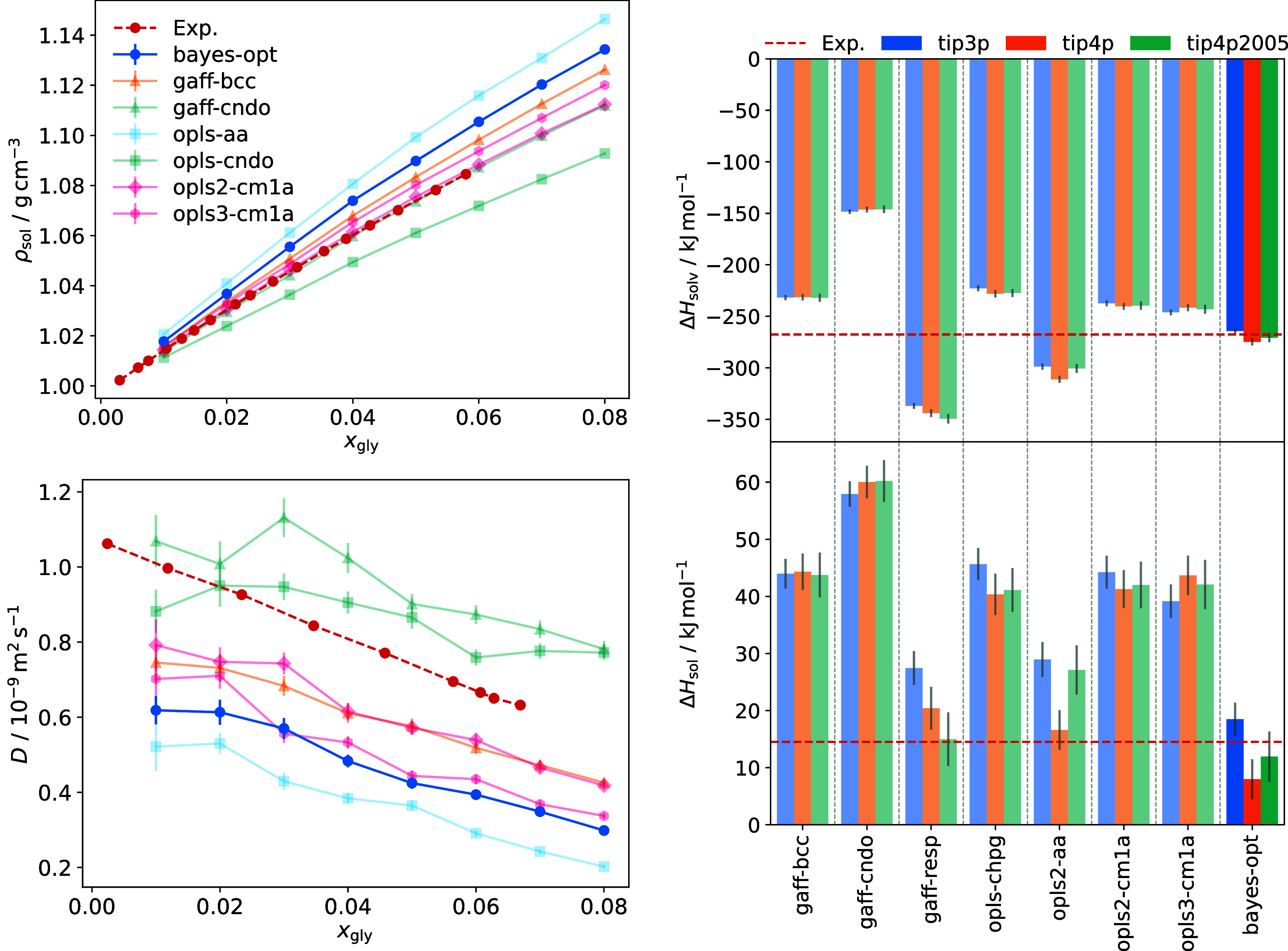
Solution-phase properties of glycine, highlighting the performance of the Bayesian optimized model compared to previously assessed force field variants. Glycine-water solution density and diffusion coefficient (left), calculated using the TIP4P/2005 water model, are compared with experimental data (red circles) from Dalton and Schmidt^70^ and Huang et al.^71^ Solvation enthalpy and α-glycine solution enthalpy values (right) are compared with experimental data (red dashed lines) from Perlovich et al.^5^

### 3.5 Analysis and Discussion

In the preceding sections we have given detailed comparisons of various unoptimized and optimized force fields in their ability to reproduce glycine crystal and solution properties, aiming to assess suitability toward investigations of crystallization and polymorphism. Our results demonstrate the inherent difficulty for simple classical force fields to simultaneously model both phases accurately. The gaff-bcc model, for example, performs strongly in reproducing static crystal and solution properties. However, mechanical instability at elevated temperatures limits its practical use for polymorph simulations.

Among unoptimized models, the opls-cm1a and opls3-cm1a force fields offer the best overall performance, combining mechanical stability, accurate lattice energies, and reasonable solution behavior. Solution enthalpy values are slightly overpredicted, indicating a preference for the solid phase, which may be advantageous for simulating crystal growth processes.

Multiobjective Bayesian optimization of the opls2-cm1a model demonstrates that targeted refinement of nonbonded parameters can reconcile relative polymorph stability while simultaneously improving crystal density and mechanical stability, albeit with a modest degradation in some solution properties. Notably, although optimization targeted only crystal-phase properties, it also improved solution enthalpy predictions to give quantitative agreement with experiment, without relying on compensating errors from poor lattice energies. This is encouraging, given that solution enthalpy is regarded as a key determinant of crystal growth behavior.^25^

Further systematic improvement of the glycine model is likely constrained by the broader limitations of standard classical force fields, which use fixed partial charges and pairwise additive potentials. Additional physics may need to be incorporated into the models — for example through polarizable force fields or machine-learned interatomic potentials,^73–75^ with advanced architectures such as MACE^76^ aiming to capture many-body effects beyond the reach of conventional pairwise models. However, these approaches would introduce additional computational overhead, which currently limits their practical use for studying crystal growth from complex systems.

The crystal property benchmarks employed here – lattice energies, crystal densities and relative polymorph stabilities – are shared with the field of crystal structure prediction (CSP), where classical force fields play a role in the initial energy ranking of candidate structures.^77,78^ However, unlike CSP, which seeks to identify stable crystal structures without prior experimental knowledge, our approach deliberately leverages experimental structures as reference data for force field calibration. The aim is to develop a model that accurately captures the structure and energetics of all three glycine polymorphs and the behavior of glycine in solution, which would therefore be capable of providing molecular insight into glycine nucleation and crystallization.

## 4 Conclusions

In this work, we have evaluated the performance of 18 parametrizations of two widely used force fields in their ability to describe the crystal and solution properties of glycine. Specifically, we considered 10 OPLS and 8 GAFF variants, each in combination with three water models. For the first time, models have been validated for all three ambient-pressure polymorphs (α-, β-, and γ-glycine).

We conclude that the best unoptimized models for simulation of glycine crystal growth are the opls-cm1a and opls3-cm1a force fields, in combination with the TIP4P/2005 water model.^32^ With methodological improvements to crystal property calculations, for α-, β- and γ-glycine, we find that these models do not underpredict the stability of crystal forms, as the widely used GAFF/CNDO model recommended by Cheong and Boon^25^ does.

Furthermore, we have demonstrated that a Bayesian optimization strategy can be effectively applied to enhance model performance in reproducing the properties tested here. Recalibrating LJ parameters improves the lattice energy ordering, unit cell densities, and mechanical stability of glycine polymorphs, while maintaining good performance for solution properties.

This optimized model will enable more accurate and reliable insights into glycine crystallization from solution and competition between its ambient-pressure polymorphs. More generally, our Bayesian optimization framework is readily extendable to other systems. This may offer a new approach for the systematic refinement of force fields for other molecular crystals and unlock a deeper understanding of polymorphism across molecular solids.

## Supplementary Material





## Notes

Glycine force field parameters; description of triclinic cell skew corrections applied to GROMACS trajectories; glycine geometries in crystal polymorphs; unit cell parameters; fitting of experimental glycine lattice parameters as a function of temperature; analysis of finite-temperature crystal stabilities; solution properties for all water model combinations; methodology for extracting diffusion coefficients from MSD curves; and convergence and phase-space overlap diagnostics for hydration free energy calculations (PDF)
Molecular topology files for all tested force fields; GROMACS simulation input files; Python analysis scripts; and tabulated data for computed crystal and solution-phase properties (ZIP)
