# Supplementary material for: Assessment and Optimization of Force Fields for Glycine Polymorphism and Solution Properties
Source: J Chem Theory Comput. 2026 Apr 7;22(8):4012–25. doi: 10.1021/acs.jctc.6c00162 (PMC13130867; doi:10.1021/acs.jctc.6c00162)
Supplement: Supplementary file 1 [file ct6c00162_si_001.pdf]

Supporting Information: Assessment and Optimisation of Force Fields for Glycine Polymorphism and Solution Properties

James W. Meadows, Sharon J. Cooper, Mark A. Miller, and Mark R. Wilson*

Department of Chemistry, Durham University, South Road, Durham DH1 3LE, UK.

E-mail: mark.wilson@durham.ac.uk

Contents

1 Glycine Force Field Parameters **S-2**

2 GROMACS Skew Correction **S-4**

3 Glycine Monomer Geometries **S-5**

4 Glycine Unit Cell Parameters **S-9**

5 Experimental Cell Parameter Fit **S-10**

6 Finite Temperature Crystal Stability **S-12**

7 Glycine Solution Properties **S-14**

8 Diffusion **S-17**

9 Hydration Free Energy Convergence **S-18**

References **S-19**

1 Glycine Force Field Parameters

Figure S1 shows the zwitterionic glycine molecule with atom labels used in this work. Point charges for these atoms are given in Table S1 for each of the charge sets tested. Partial charges vary significantly across the different sets, particularly for the ammonium nitrogen (N) and alpha carbon (CA). Tables S2 and S3 give the LJ parameters for glycine atom types in the OPLS and GAFF force field variants tested in this work, respectively.

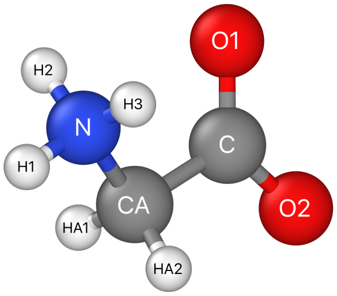

Figure S1: Zwitterionic glycine molecule with atom labels.

Table S1: Atomic charge sets.

Atom	aa	chpg	cm1a	cndo	bcc	dnp	resp	abcg2
N	−0.300	−0.352	−0.4549	0.022	−0.8356	−0.127	−0.1697	−0.9730
H1	0.330	0.317	0.3940	0.164	0.4438	0.199	0.2957	0.4990
H2	0.330	0.317	0.3940	0.208	0.4438	0.218	0.2957	0.4990
H3	0.330	0.317	0.3940	0.199	0.4438	0.224	0.2957	0.4990
CA	0.090	−0.029	−0.2299	−0.021	−0.0952	0.007	0.0171	−0.1234
HA1	0.060	0.074	0.1343	0.033	0.0887	0.064	0.0711	0.0887
HA2	0.060	0.074	0.1343	0.030	0.0887	0.062	0.0711	0.0887
C	0.700	0.700	0.5018	0.374	0.9326	0.483	0.8951	0.7220
O1	−0.800	−0.709	−0.6338	−0.526	−0.7553	−0.578	−0.8859	−0.6500
O2	−0.800	−0.709	−0.6338	−0.483	−0.7553	−0.552	−0.8859	−0.6500

Table S2: LJ parameters of glycine atom types for the OPLS force field variants.

	opls		opls2		opls3	
Atom	σ / Å	ϵ / kJ mol^{-1}	σ / Å	ϵ / kJ mol^{-1}	σ / Å	ϵ / kJ mol^{-1}
N	3.250 00	0.711 280	3.480 00	1.213 360	3.250 00	0.711 280
H	0.000 00	0.000 000	0.000 00	0.000 000	0.000 00	0.000 000
CA	3.500 00	0.276 144	3.510 00	0.276 144	3.500 00	0.276 144
HA	2.500 00	0.125 520	2.480 00	0.108 784	2.500 00	0.125 520
C	3.750 00	0.439 320	3.750 00	0.439 320	3.550 00	0.292 880
O	2.960 00	0.878 640	2.960 00	0.878 640	2.960 00	0.878 640

Table S3: LJ parameters of glycine atom types for the GAFF force field variants.

	gaff		gaff2	
Atom	σ / Å	ϵ / kJ mol^{-1}	σ / Å	ϵ / kJ mol^{-1}
N	3.2500	0.711 28	2.7668	4.790 68
H	1.0691	0.065 69	1.1065	0.041 84
CA	3.3997	0.457 73	3.3977	0.451 04
HA	1.9600	0.065 69	1.8875	0.087 03
C	3.4000	0.359 82	3.3152	0.413 38
O	2.9599	0.878 64	3.0481	0.612 12

2 GROMACS Skew Correction

GROMACS 2022.3[S1,S2] represents the triclinic box with 3 vectors

$$\mathbf{a} = \begin{pmatrix} a_x \\ 0 \\ 0 \end{pmatrix}, \quad \mathbf{b} = \begin{pmatrix} b_x \\ b_y \\ 0 \end{pmatrix}, \quad \mathbf{c} = \begin{pmatrix} c_x \\ c_y \\ c_z \end{pmatrix} \tag{1}$$

with constraints on their components that prevent the box from becoming too skewed:

$$|b_x| \leq \frac{1}{2}a_x, \quad |c_x| \leq \frac{1}{2}a_x, \quad |c_y| \leq \frac{1}{2}b_y. \tag{2}$$

These constraints are satisfied through the addition and subtraction of box vectors to yield equivalent representations of the system. However, these transformations create sudden jumps in cell parameters during the simulation. For example, if $\mathbf{a} = (5, 0, 0)\,\text{nm}$ and $\mathbf{b} = (3, 1, 0)\,\text{nm}$ GROMACS will subtract $\mathbf{a}$ from $\mathbf{b}$, giving $\mathbf{b} = (-2, 1, 0)\,\text{nm}$. Average cell parameters are computed by first averaging the components of $\mathbf{a}$, $\mathbf{b}$ and $\mathbf{c}$. Therefore, to obtain the correct averages, we must have a consistent triclinic cell representation throughout the simulation trajectory. For each configuration i, we transform the triclinic vectors by comparing component values with those from the preceding configuration $i-1$

$$\begin{aligned} \mathbf{b}^{(i)} &\leftarrow \mathbf{b}^{(i)} - \text{round}\left(\frac{b_x^{(i)} - b_x^{(i-1)}}{a_x^{(i)}}\right) \mathbf{a}^{(i)} \\ \mathbf{c}^{(i)} &\leftarrow \mathbf{c}^{(i)} - \text{round}\left(\frac{c_x^{(i)} - c_x^{(i-1)}}{a_x^{(i)}}\right) \mathbf{a}^{(i)} \\ \mathbf{c}^{(i)} &\leftarrow \mathbf{c}^{(i)} - \text{round}\left(\frac{c_y^{(i)} - c_y^{(i-1)}}{b_y^{(i)}}\right) \mathbf{b}^{(i)}. \end{aligned} \tag{3}$$

Note that $\mathbf{b}$ is transformed first as it is used later in the transformation of $\mathbf{c}$.

3 Glycine Monomer Geometries

Tables S4, S5 and S6 describe the molecular geometries of glycine monomers in the α-, β- and γ-glycine polymorphs, respectively, as predicted by the opls-cm1a, opls3-cm1a and bayes-opt force fields. Experimental values are also given for comparison, calculated for Cambridge Structural Database (CSD) entries GLYCIN98,[S3] GLYCIN71,[S4] and GLYCIN33[S5] at temperatures of 10 K, 293 K and 294 K, respectively. Root mean squared deviation (RMSD) values of atomic positions are given for each force field, calculated with respect to the experimental geometry.

For α- and β-glycine, all force fields give RMSD values around 0.2 Å, indicating good reproduction of the experimental monomer geometry. Larger deviations for γ-glycine are primarily driven by a rotation of the carboxylate group. The tested force fields impose weak or absent torsional barriers around the CA-C bond, so the orientation of the carboxylate plane is governed primarily by non-bonded interactions with the crystal environment. Such deviations are therefore not unexpected for flexible fixed-charge force fields, where the monomer geometry will be strongly coupled to crystal packing and sensitive to the local electrostatic environment of each polymorph.

Table S4: Glycine monomer geometry in α-glycine for the opls-cm1a, opls3-cm1a and bayes-opt force fields, compared with experimental values from CSD entry GLYCIN98.[S3]

	opls-cm1a	opls3-cm1a	bayes-opt	Exp.
		Bonds: r_{ij} / Å		
N-H1	1.032	1.031	1.027	1.004
N-H2	1.026	1.026	1.023	0.956
N-H3	1.023	1.023	1.021	0.835
N-CA	1.471	1.471	1.469	1.485
CA-HA1	1.090	1.090	1.090	1.012
CA-HA2	1.089	1.088	1.093	0.937
CA-C	1.508	1.509	1.510	1.529
C-O1	1.246	1.247	1.250	1.251
C-O2	1.246	1.245	1.247	1.264
		Angles: θ_{ijk} / deg		
N-CA-HA1	109.7	109.7	109.9	107.1
N-CA-HA2	110.1	110.2	110.6	108.0
N-CA-C	108.2	108.3	109.0	111.2
H1-N-H2	108.9	109.6	110.5	106.7
H1-N-H3	109.3	109.9	109.3	106.2
H1-N-CA	113.5	113.6	113.8	111.8
H2-N-H3	106.5	107.3	105.4	114.6
H2-N-CA	109.2	108.3	109.3	108.3
H3-N-CA	109.2	107.8	108.2	109.3
CA-C-O1	117.5	117.6	118.0	117.7
CA-C-O2	118.5	118.4	117.7	116.4
HA1-CA-HA2	107.9	107.9	107.5	112.7
HA1-CA-C	110.6	110.4	109.6	109.0
HA2-CA-C	110.4	110.2	110.2	109.0
O1-C-O2	123.6	123.7	124.1	125.9
		Dihedrals: ϕ_{ijkl} / deg		
H1-N-CA-HA1	−54.4	−54.0	−53.4	−56.8
H1-N-CA-HA2	64.3	64.7	65.0	64.8
H1-N-CA-C	−175.0	−174.6	−173.6	−175.7
H2-N-CA-HA1	−176.0	−176.0	−177.4	−174.0
H2-N-CA-HA2	−57.4	−57.3	−59.0	−52.5
H2-N-CA-C	63.3	63.4	62.3	67.0
H3-N-CA-HA1	67.9	68.1	68.3	60.5
H3-N-CA-HA2	−173.5	−173.2	−173.3	−178.0
H3-N-CA-C	−52.8	−52.5	−51.9	−58.4
N-CA-C-O1	−39.4	−35.4	−33.6	−19.6
N-CA-C-O2	132.7	138.3	142.0	161.5
HA1-CA-C-O1	−159.6	−155.6	−153.9	−137.4
HA1-CA-C-O2	12.6	18.2	21.7	43.7
HA2-CA-C-O1	81.0	85.3	88.0	99.3
HA2-CA-C-O2	−106.8	−101.0	−96.4	−79.6
		RMSD / Å		
	0.22	0.19	0.17	—

Table S5: Glycine monomer geometry in β-glycine for the opls-cm1a, opls3-cm1a and bayes-opt force fields, compared with experimental values from CSD entry GLYCIN71.[S4]

	opls-cm1a	opls3-cm1a	bayes-opt	Exp.
Bonds: r_{ij} / Å				
N-H1	1.027	1.027	1.025	1.008
N-H2	1.023	1.024	1.021	1.002
N-H3	1.025	1.025	1.024	1.009
N-CA	1.469	1.470	1.469	1.495
CA-HA1	1.089	1.088	1.091	0.987
CA-HA2	1.089	1.089	1.092	0.977
CA-C	1.507	1.507	1.507	1.602
C-O1	1.247	1.247	1.249	1.239
C-O2	1.246	1.246	1.246	1.224
Angles: θ_{ijk} / deg				
N-CA-HA1	109.2	109.1	109.9	105.3
N-CA-HA2	109.4	109.4	110.2	106.4
N-CA-C	109.4	109.4	109.5	112.6
H1-N-H2	111.9	112.3	110.7	109.1
H1-N-H3	108.4	109.5	108.8	110.5
H1-N-CA	110.1	109.7	111.9	109.9
H2-N-H3	108.3	108.9	107.2	108.4
H2-N-CA	109.8	108.7	109.5	110.0
H3-N-CA	108.2	107.7	108.6	108.8
CA-C-O1	117.4	117.4	118.0	116.7
CA-C-O2	117.8	117.8	117.5	116.7
HA1-CA-HA2	107.8	107.9	107.7	109.9
HA1-CA-C	110.5	110.5	108.7	110.2
HA2-CA-C	110.6	110.5	110.7	112.1
O1-C-O2	124.7	124.7	124.5	126.4
Dihedrals: ϕ_{ijkl} / deg				
H1-N-CA-HA1	−53.9	−54.3	−53.0	−73.4
H1-N-CA-HA2	63.8	63.6	65.6	43.3
H1-N-CA-C	−175.0	−175.3	−172.4	166.5
H2-N-CA-HA1	−177.6	−177.4	−176.1	166.5
H2-N-CA-HA2	−59.8	−59.6	−57.5	−76.9
H2-N-CA-C	61.4	61.6	64.5	46.3
H3-N-CA-HA1	64.4	64.8	67.1	47.8
H3-N-CA-HA2	−177.8	−177.4	−174.4	164.5
H3-N-CA-C	−56.6	−56.3	−52.3	−72.3
N-CA-C-O1	32.9	32.1	33.5	19.5
N-CA-C-O2	−145.1	−146.1	−145.6	−165.8
HA1-CA-C-O1	−87.4	−88.1	−86.6	−97.8
HA1-CA-C-O2	94.7	93.7	94.2	76.9
HA2-CA-C-O1	153.4	152.6	155.3	139.4
HA2-CA-C-O2	−24.6	−25.6	−23.9	−45.9
RMSD / Å				
	0.22	0.21	0.24	—

Table S6: Glycine monomer geometry in γ-glycine for the opls-cm1a, opls3-cm1a and bayes-opt force fields, compared with experimental values from CSD entry GLYCIN33.[S5]

	opls-cm1a	opls3-cm1a	bayes-opt	Exp.
		Bonds: r_{ij} / Å		
N-H1	1.024	1.024	1.021	0.888
N-H2	1.024	1.024	1.021	0.899
N-H3	1.031	1.030	1.023	0.887
N-CA	1.473	1.473	1.474	1.462
CA-HA1	1.091	1.091	1.092	0.969
CA-HA2	1.088	1.089	1.091	0.970
CA-C	1.506	1.506	1.509	1.532
C-O1	1.242	1.242	1.245	1.260
C-O2	1.247	1.247	1.247	1.238
		Angles: θ_{ijk} / deg		
N-CA-HA1	109.9	109.9	110.9	109.8
N-CA-HA2	110.1	110.1	109.8	109.2
N-CA-C	109.1	109.0	110.5	111.9
H1-N-H2	110.1	110.3	109.5	109.3
H1-N-H3	104.1	105.7	105.9	109.2
H1-N-CA	112.7	112.0	112.9	109.9
H2-N-H3	105.5	106.7	104.8	109.4
H2-N-CA	112.0	110.8	110.7	109.6
H3-N-CA	111.9	111.1	112.6	109.3
CA-C-O1	118.8	118.7	117.7	117.1
CA-C-O2	117.4	117.4	118.3	117.0
HA1-CA-HA2	107.3	107.5	107.5	107.8
HA1-CA-C	111.0	111.0	110.1	109.2
HA2-CA-C	109.5	109.4	108.0	108.9
O1-C-O2	123.8	124.0	123.9	125.9
		Dihedrals: ϕ_{ijkl} / deg		
H1-N-CA-HA1	−74.7	−74.7	−67.8	−62.7
H1-N-CA-HA2	43.2	43.5	50.8	55.3
H1-N-CA-C	163.4	163.5	169.9	176.0
H2-N-CA-HA1	160.5	161.7	169.1	177.1
H2-N-CA-HA2	−81.6	−80.2	−72.2	−64.9
H2-N-CA-C	38.6	39.9	46.8	55.7
H3-N-CA-HA1	42.3	43.2	52.1	57.3
H3-N-CA-HA2	160.2	161.4	170.8	175.3
H3-N-CA-C	−79.6	−78.6	−70.2	−64.1
N-CA-C-O1	132.6	130.3	152.0	15.8
N-CA-C-O2	−46.6	−48.6	−27.7	−167.3
HA1-CA-C-O1	11.4	9.2	29.2	−106.0
HA1-CA-C-O2	−167.8	−169.7	−150.4	71.0
HA2-CA-C-O1	−106.9	−109.2	−88.0	136.6
HA2-CA-C-O2	73.9	71.9	92.4	−46.5
		RMSD / Å		
	0.82	0.81	0.90	—

4 Glycine Unit Cell Parameters

Table S7 shows the unit cell parameters for glycine polymorphs at 0 K, as predicted by the opls-cm1a, opls3-cm1a and bayes-opt force fields. Unit cell parameters for the other tested force fields are given in the supplementary data.

Table S7: Unit cell parameters for glycine polymorphs, for the opls-cm1a, opls3-cm1a and bayes-opt force fields. Experimental reference data are obtained from a fit of crystallographic measurements, described in Section 5. Cell lengths a, b and c are given in Å, angles α, β and γ are given in °, and cell volumes V are given in Å^3.

	a	b	c	α	β	γ	V
				α-glycine			
opls-cm1a	4.99	12.90	5.28	90.0	112.2	90.0	314.9
opls3-cm1a	4.98	12.61	5.28	90.0	112.4	90.0	306.1
bayes-opt	4.99	12.06	5.39	90.0	109.1	90.0	306.6
Exp.	5.08	11.77	5.46	90.0	112.1	90.0	302.8
				β-glycine			
opls-cm1a	5.16	6.68	4.90	90.0	113.4	90.0	155.0
opls3-cm1a	5.16	6.55	4.90	90.0	113.1	90.0	152.1
bayes-opt	5.29	6.58	4.95	90.0	114.4	90.0	157.0
Exp.	5.07	6.14	5.39	90.0	113.5	90.0	154.0
				γ-glycine			
opls-cm1a	7.03	7.03	5.32	90.0	90.0	120.0	245.1
opls3-cm1a	7.29	7.29	5.30	90.0	90.0	120.0	243.8
bayes-opt	6.92	6.92	5.67	90.0	90.0	120.0	236.2
Exp.	6.97	6.97	5.48	90.0	90.0	120.0	230.3

5 Experimental Cell Parameter Fit

Zero-temperature reference data for the unit cell parameters of glycine polymorphs were obtained by fitting crystallographic measurements from Boldyreva et al.[S5] Lattice parameters were modelled quadratically as a function of temperature. For example, the a parameter was fitted as

$$a(T) = a_0 + a_1 T + a_2 T^2, \tag{4}$$

with analogous expressions used for b, c, and, for monoclinic polymorphs, β. Coefficients were optimised using nonlinear least-squares minimisation such that the unit cell volume

$$V(T) = a(T)b(T)c(T)\sqrt{1 + 2\cos\alpha\cos\beta(T)\cos\gamma - \cos^2\alpha - \cos^2\beta(T) - \cos^2\gamma} \tag{5}$$

remained internally consistent with the fitted lattice parameters at each temperature. Figure S2 shows the volume per molecule obtained from these fitted curves, along with additional data from the CSD. Similar plots are shown for the individual lattice parameters in Figure S3.

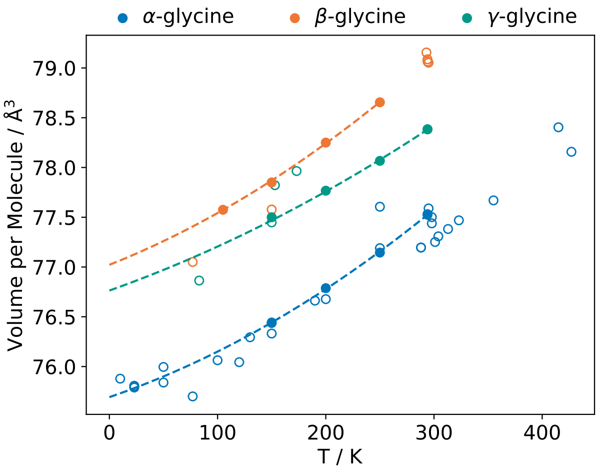

Figure S2: Volume per molecule for glycine ambient pressure polymorphs. Solid circles represent data from Boldyreva et al.,[S5] used in the quadratic fit. Open circles represent data from CSD entries.

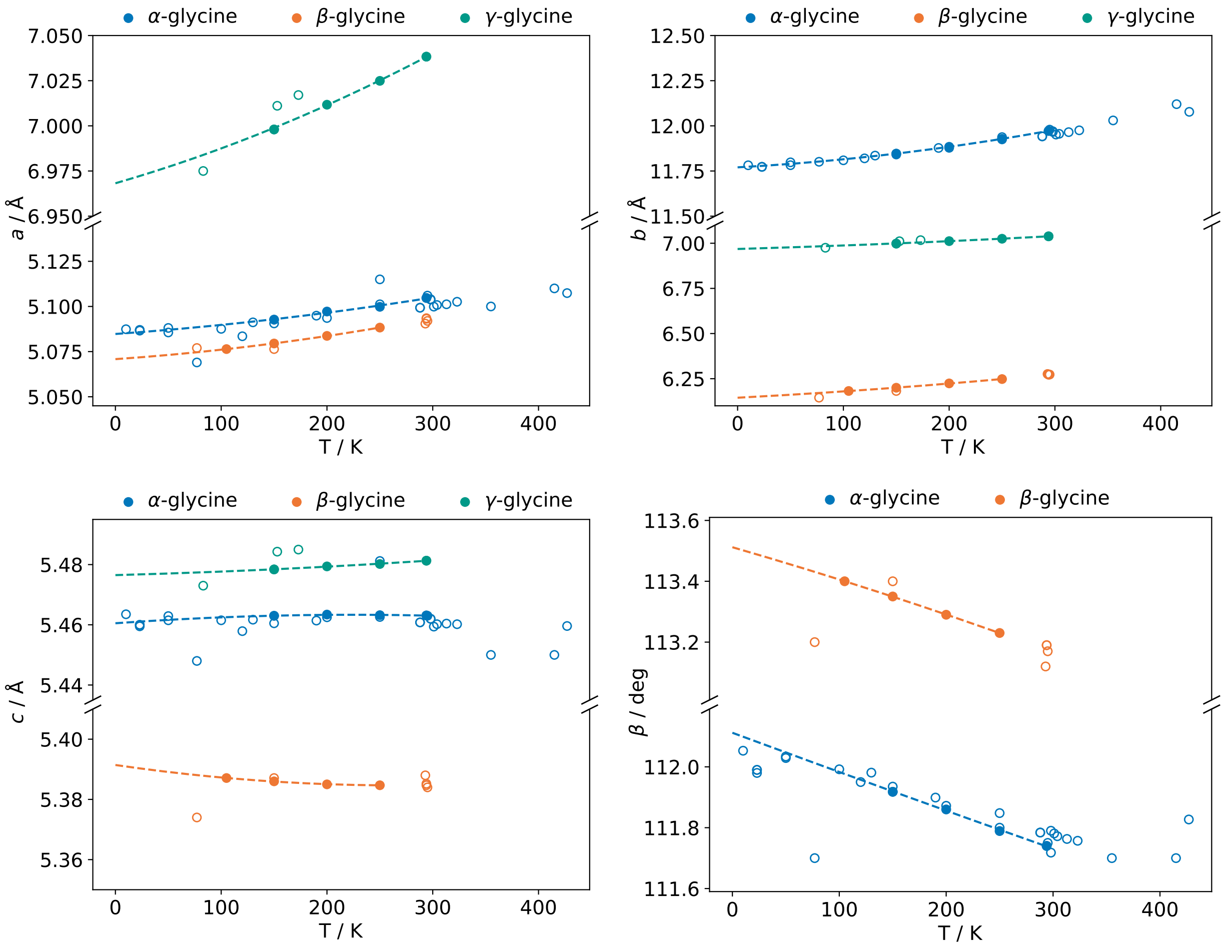

Figure S3: Lattice parameters for glycine ambient pressure polymorphs. Solid circles represent data from Boldyreva et al.,[S5] used in the quadratic fits. Open circles represent data from CSD entries.

6 Finite Temperature Crystal Stability

Figure S4 shows the reduction in crystal density during equilibration for α-glycine modelled with gaff-bcc and β-glycine modelled with gaff-cndo. Supercells were equilibrated carefully, increasing temperature from 50 K to a target value over a 4 ns simulation. For the gaff-bcc model, the crystal supercell exhibits a sharp density decrease when increasing temperature causes the system to cross a threshold of approximately $1620\,\mathrm{kg\,m^{-3}}$. At temperatures above 150 K, the density of β-glycine modelled with the gaff-cndo force field also exhibits unphysical behaviour.

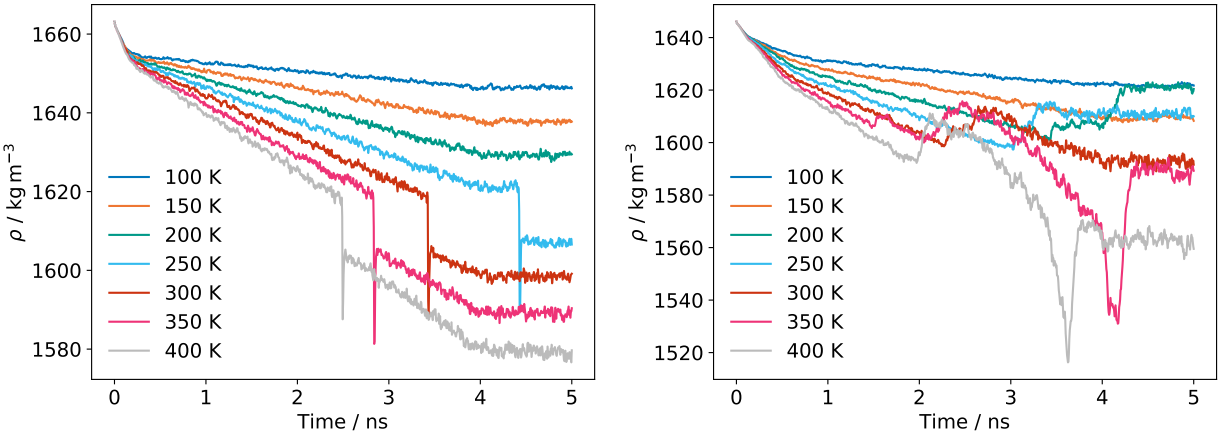

Figure S4: Crystal density of α-glycine using gaff-bcc (left) and β-glycine using gaff-cndo (right) during equilibration with temperature increasing from 50 K to a target value.

Conversely, the opls-cm1a and opls3-cm1a variants are mechanically stable for all three polymorphs up to 400 K. Figure S5 shows the behaviour of the opls3-cm1a model across this temperature range. Crystal densities vary smoothly during equilibration. After equilibration, average unit cell parameters were calculated from an additional 5 ns simulation and used to determine the volume per glycine molecule at each temperature. Slopes of the experimental curves are captured well, although relative volumes for each polymorph are ordered incorrectly. For the α, β and γ forms, the maximum deviations from experimental cell densities across the entire temperature range are −3.4%, 0.9% and −5.6% respectively.

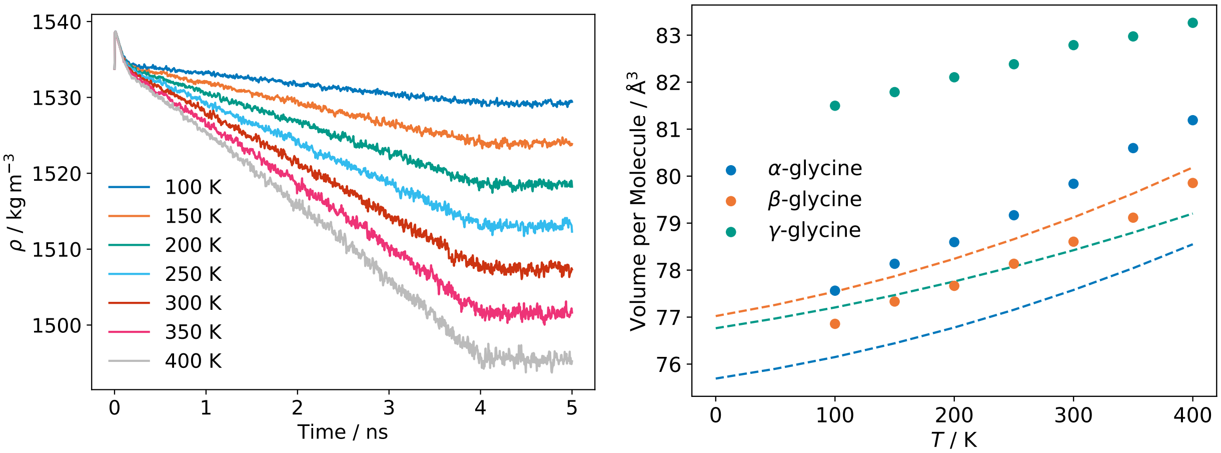

Figure S5: Crystal density of γ-glycine (left) during equilibration and thermal expansion of polymorphs (right) modelled using the opls3-cm1a force field. Dashed lines show experimental values obtained from a fit of crystallographic measurements at various temperatures.[S5]

7 Glycine Solution Properties

Figures S6 and S7 show solution density and glycine diffusion curves as a function of glycine mole fraction, for all tested force fields and water models.

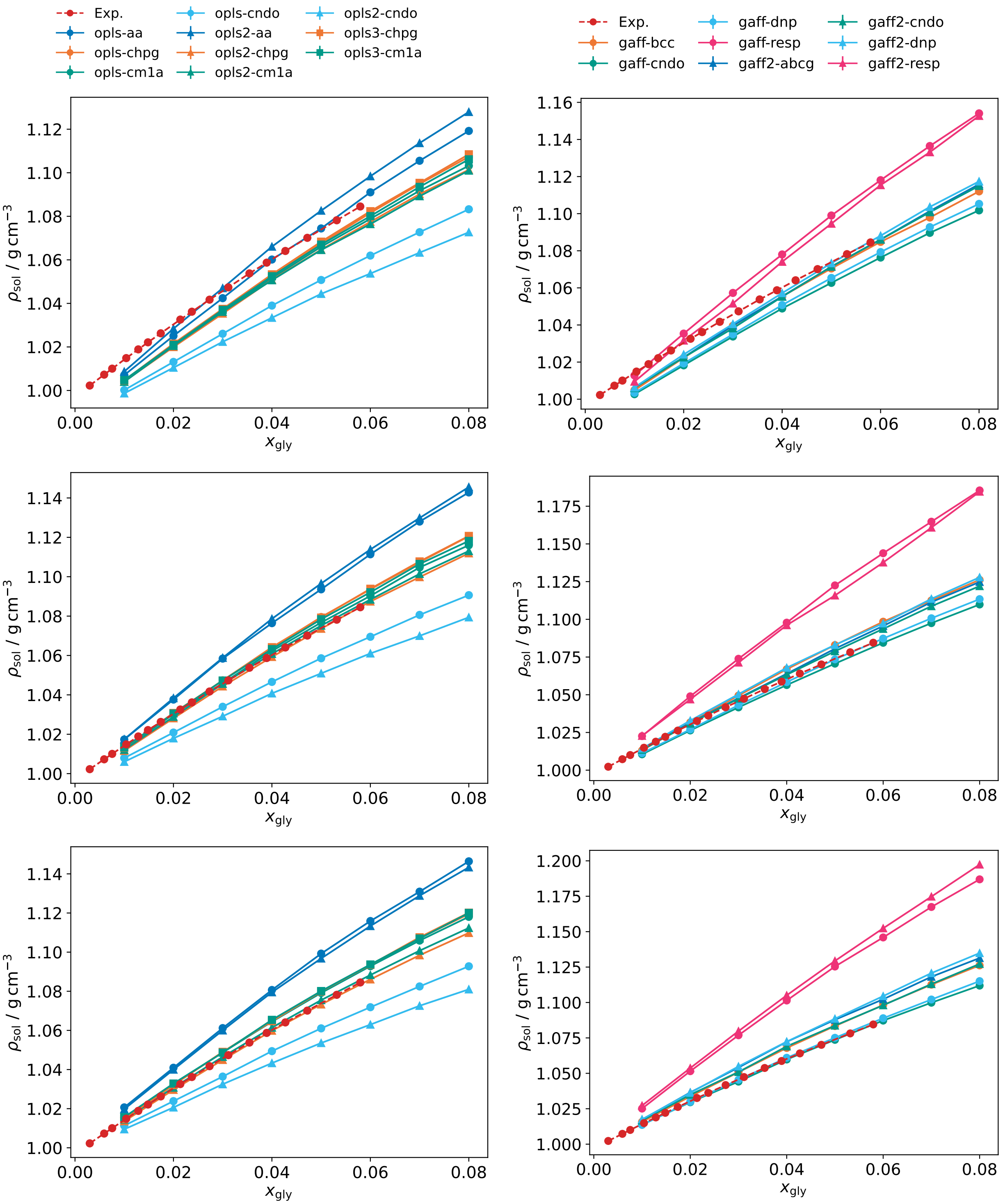

Figure S6: Density of glycine-water solution as a function of glycine mole fraction for TIP3P (top), TIP4P (middle), and TIP4P/2005 (bottom) water models, compared with experimental data (red circles) from Dalton and Schmidt.[S6] Lines with the same colour utilise the same charge set, and lines with the same marker shape have the same non-bonded parameters.

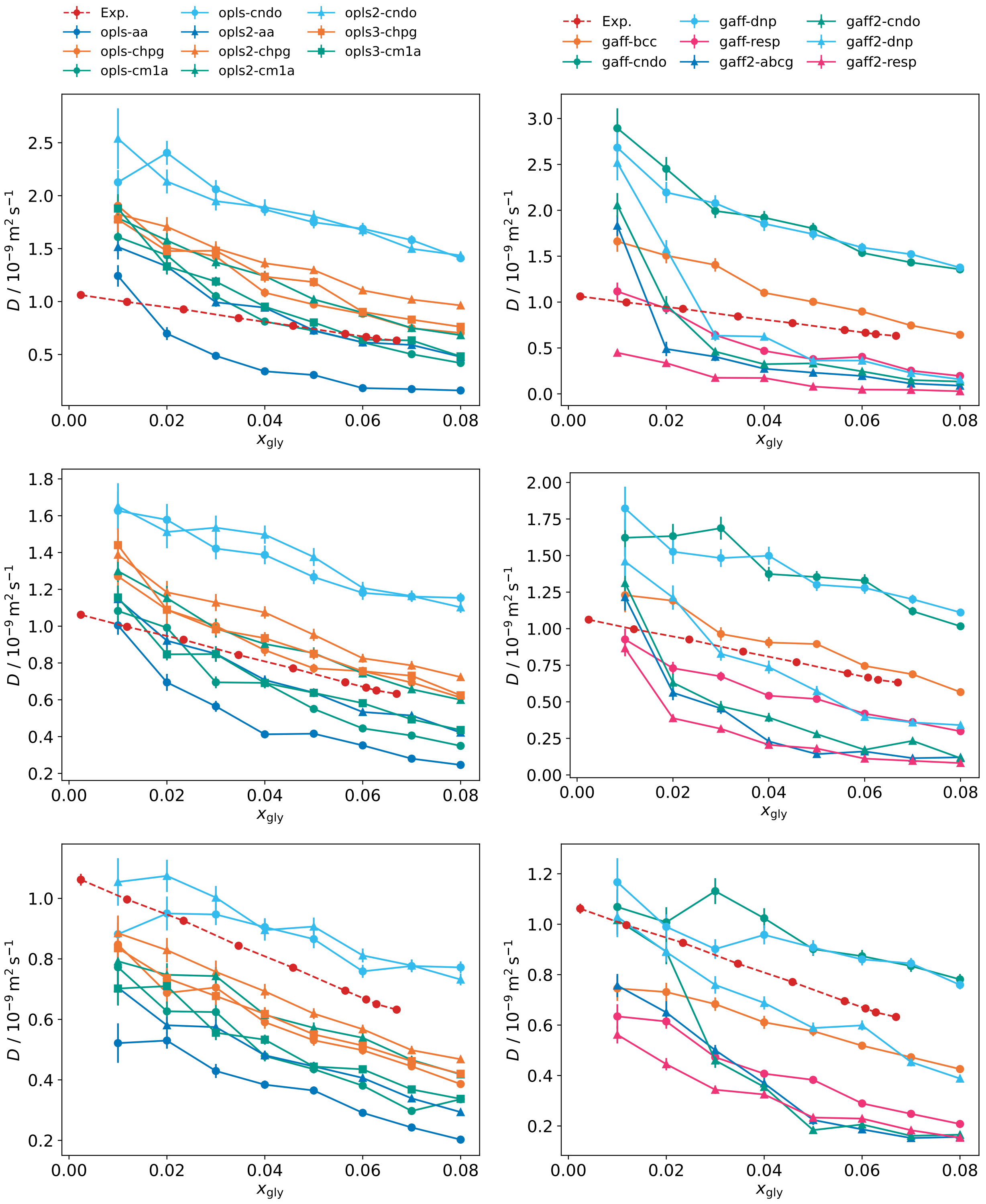

Figure S7: Glycine diffusion coefficient as a function of mole fraction for TIP3P (top), TIP4P (middle), and TIP4P/2005 (bottom) water models, compared with experimental data (red circles) from Huang et al..[S7] Lines with the same colour utilise the same charge set, and lines with the same marker shape have the same non-bonded parameters.

8 Diffusion

Figure S8 shows representative curves for the mean squared displacement (MSD) of glycine in water, in this case for the opls2-cm1a model with TIP4P/2005 water. To derive the diffusion coefficient, we take the slope of the MSD curve up to $\tau = 2\,\text{ns}$, where the diffusion exponent

$$\alpha(\tau) = \frac{d \log \langle \Delta \mathbf{r}^2(\tau) \rangle}{d \log \tau} \tag{6}$$

is close to 1.0 and we are in the diffusive regime.

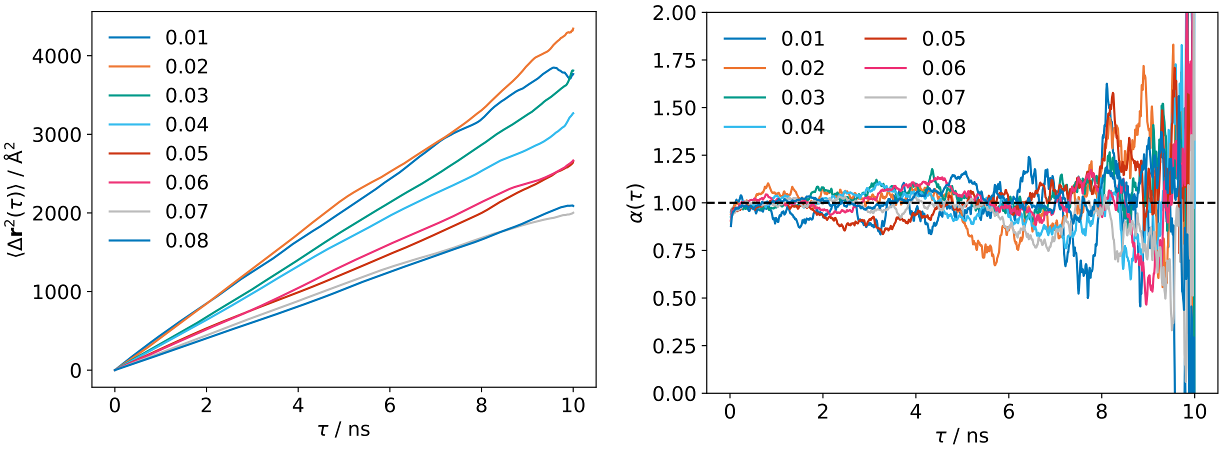

Figure S8: MSD vs lagtime (left) for glycine in solution with mole fractions x_{gly} ranging from 0.01 to 0.08. Diffusion exponent $\alpha(\tau)$ vs lagtime (right) for the MSD curves.

9 Hydration Free Energy Convergence

Figure S9 shows example convergence plots for the opls-aa force field in TIP4P/2005 water at 298.15 K. Poor equilibration can be identified by plotting the free energy estimate computed with an increasing fraction of the simulation data, for both the forward and backward time series.[S8] In this case, both the forward and backward estimates agree within error after around a third of the total simulation time, which indicates good convergence.

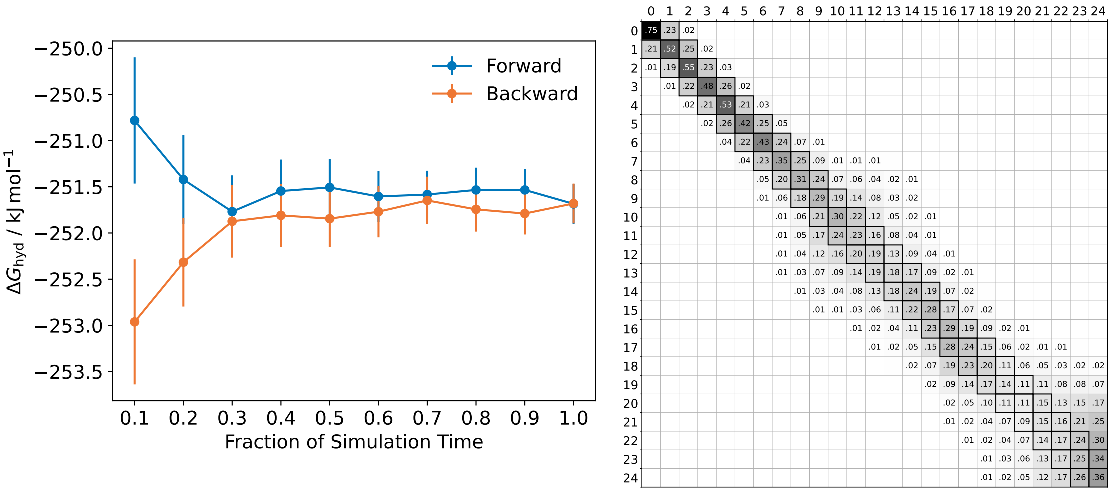

Figure S9: Hydration free energy time convergence (left) and MBAR overlap matrix (right) for the opls-aa force field in TIP4P/2005 water at 298.15 K.

Accurate free energy estimation with the MBAR estimator requires good phase-space overlap between λ points. We can assess this overlap by computing the overlap matrix, where each element O_{ij} represents the probability of finding a sample from state i (row) in state j (column). For example, Figure S9 shows that the probability of finding a sample from $\lambda = 4$ in state $\lambda = 5$ is 0.21. Klimovich et al.[S8] suggest that this matrix should be tridiagonal for reliable free energy estimates, with the probability of overlap between neighbouring λ points at least 0.03. The overlap shown here is more than sufficient for an accurate estimate of ΔG_{hyd}.

References

(S1) Bekker, H.; Berendsen, HJC.; Dijkstra, EJ.; Achterop, S.; Vondrumen, R.; Vanderspoel, D.; Sijbers, A.; Keegstra, H.; Renardus, MKR. GROMACS - A Parallel Computer for Molecular-Dynamics Simulations: 4th International Conference on Computational Physics (PC 92). *Physics Computing '92* **1993**, 252–256.

(S2) Abraham, M. J.; Murtola, T.; Schulz, R.; Páll, S.; Smith, J. C.; Hess, B.; Lindahl, E. GROMACS: High Performance Molecular Simulations through Multi-Level Parallelism from Laptops to Supercomputers. *SoftwareX* **2015**, *1–2*, 19–25.

(S3) Aree, T.; Bürgi, H.-B. Dynamics and Thermodynamics of Crystalline Polymorphs: α-Glycine, Analysis of Variable-Temperature Atomic Displacement Parameters. *The Journal of Physical Chemistry A* **2012**, *116*, 8092–8099.

(S4) Tumanov, N. A.; Boldyreva, E. V.; Ahsbahs, H. Structure Solution and Refinement from Powder or Single-Crystal Diffraction Data? Pros and Cons: An Example of the High-Pressure β'-Polymorph of Glycine. *Powder Diffraction* **2008**, *23*, 307–316.

(S5) Boldyreva, E. V.; Drebushchak, T. N.; Shutova, E. S. Structural Distortion of the α, β, and γ Polymorphs of Glycine on Cooling. *Zeitschrift für Kristallographie - Crystalline Materials* **2003**, *218*, 366–376.

(S6) Dalton, J. B.; Schmidt, C. L. A. The Solubilities of Certain Amino Acids in Water, the Densities of Their Solutions at Twenty-Five Degrees, and the Calculated Heats of Solution and Partial Molal Volumes. *Journal of Biological Chemistry* **1933**, *103*, 549–578.

(S7) Huang, J.; Stringfellow, T. C.; Yu, L. Glycine Exists Mainly as Monomers, Not Dimers, in Supersaturated Aqueous Solutions: Implications for Understanding Its Crystallization and Polymorphism. *Journal of the American Chemical Society* **2008**, *130*, 13973–13980.

(S8) Klimovich, P. V.; Shirts, M. R.; Mobley, D. L. Guidelines for the Analysis of Free Energy Calculations. *Journal of Computer-Aided Molecular Design* **2015**, *29*, 397–411.
